# Supplementary material for: “It’s my calling”, Canadian dog rescuers’ motives and experiences for engaging in international dog rescue efforts
Source: PLoS One. 2024 May 31;19(5):e0300104. doi: 10.1371/journal.pone.0300104 (PMC11142615; doi:10.1371/journal.pone.0300104)
Supplement: S1 File — (DOCX) [file pone.0300104.s001.docx]

Dear [Rescue Organization],

I am Kai, a master’s student in the Applied Animal Biology Program at the University of British Columbia. I’m writing to you today in my role as a Co-Investigator of a study interviewing rescue organization professionals for their perspectives, attitudes, and experiences gained through years of working in this field. The goal of this research is to support foreign dog adoption by gaining a better understanding of the rescue process directly from the people involved in foreign dog rescue.

In order to meet these goals, the current study will be recruiting 10 rescue organization professionals to participate in a 60-minute interview. Alexandra Protopopova (Principal Investigator) and I recognize you as an expert in your field and would value the opportunity to learn from you, if you decide to participate. Specifically, we will ask you about the motives (primary goals) of your rescue organization, commonly faced hardships in your line of work, and the most rewarding part of your work. We will not ask questions of a personal nature or any specific questions that may disrepute your organization. You may also refuse to answer questions and end the interview at any point without consequences. The identity of you and your organization will not be disclosed at any point in this research. Additionally, all identifiable information will be removed in any publication or reporting of the current research findings. Any data related to this research will be encrypted and stored in a password-protected computer of the study team.

Due to the Covid-19 pandemic, we will be commencing the interview virtually through the Zoom software. With your consent, we would like to audio-record the interview to preserve accuracy of content until we are able to transcribe the interview for analysis.

Participation in this interview is entirely voluntary. There are no compensations for participating.

We will conduct the interview during September and will be able to accommodate your schedule. Please respond to this email if you are interested in participating in this research. Additionally, please also feel free to contact me if you have any questions or concerns regarding what we are asking of you.

Thank you for your consideration, and we wish you all the very best in your work.

Sincerely,

Alexandra Protopopova, PhD

Principal Investigator

Assistant Professor at UBC, Faculty of Land and Food Systems

Animal Welfare Program

Kai von Rentzell, MSc

Co-Investigator

Graduate Student at UBC, Faculty of Land and Food Systems

Animal Welfare Program
