# Supplementary material for: “It’s my calling”, Canadian dog rescuers’ motives and experiences for engaging in international dog rescue efforts
Source: PLoS One. 2024 May 31;19(5):e0300104. doi: 10.1371/journal.pone.0300104 (PMC11142615; doi:10.1371/journal.pone.0300104)
Supplement: S2 File — (DOCX) [file pone.0300104.s002.docx]

**Dog Rescue Organization Semi-Structured Interview Guide**

**Introductory Statements:**

Purpose: This interview is part of a research study aimed to improve our understanding about the importation of dogs to British Columbia with a purpose of adoption into pet homes. This interview will highlight the perspectives and experiences of the rescue organizations that import dogs for adoption; these perspectives are currently not included in the scientific literature. Specifically, we are interested in learning about the primary goals and challenges of rescue organizations that import dogs into British Columbia.

Researcher Team: This research is carried out by me, Kai von Rentzell, a Master’s student in the Animal Welfare Program at The University of British Columbia, and my supervisor, Dr. Alexandra (Sasha) Protopopova, an Assistant Professor also in the Animal Welfare Program at the University of British Columbia.

Interview Duration: The interview will not be longer than 60 minutes.

Assurance of Confidentiality: As you have read in the consent form, your identity will not be disclosed at any point in this research. The name of you or your organization will not be revealed in any publication or reports of the findings of the current study, and interview transcripts will not contain any identifiable information. This will ensure that both your participation in the interview as well as your responses are confidential. During the data analysis process, the collected data will be encrypted and stored in a password-locked computer of the research team.

Digital Recording: With your permission, I would like to record this interview to preserve the accuracy of its content. These recordings will be kept confidential and will only be accessed by the research team. These recordings will only be used for the purpose of the creation of interview transcripts and will be deleted following transcription.

Additional Assurance: Please do not hesitate to ask for clarification if any of the questions I ask are unclear. Additionally, your participation in this interview is entirely voluntary. You are welcome to decline answering questions and you may ask to stop the interview at any point. If you have questions for me, please feel free to ask at any time.

As I ask you various questions, please remember that there are no right or wrong answers. We only want your unique perspective. You have years of rich experience working in this unique profession, and I would find it most helpful if you could speak from this place of experience. Feel free to share specific examples if that’s helpful to your answer. I’m looking forward to being in dialogue and learning from you.

**Terms and Definitions:**

Before we begin, I wanted to clarify some terms as I use them.

Source Shelter: Collaborating shelters that are in the country of origin from where dogs are rescued.

Source Community: Collection of shelters, rescues, people in the country of origin from where dogs are rescued.

Destination: The final destination of the rescued dog- in our case, our community here in Lower mainland of BC, Canada.

**Questions**

**Introductory question:** Will you please remind me, from which geographic areas does your rescue organization import dogs?

| **Research Question:**  *What are the* *motives* *of rescue organizations that import foreign rescue dogs?*  *What are common challenges faced by rescue organizations?* | |
| --- | --- |
| **Question 1:** Tell me about how your organization came to the decision to rescue dogs from [geographical area]? | |
| **Follow-ups:**   - Was your rescue organization originally focused on local adoptions?   - If yes: How did your rescue transition to non-Canadian adoptions?  - If no: Why your rescue targeted foreign adoptions at the outset.   - Please elaborate on how you got involved with source shelter/fosters? | **Notes:** |
| **Question 2:** Tell me about the factors you consider when selecting dogs to bring over from [geographical area]? For example, do you consider the dog’s adoptability in BC, the cost that would be involved for their transport, or do you bring dogs upon request by shelters you are working with? | |
| **Follow-ups:**   - Please describe to me a ‘typical’ dog you rescue? - Please describe to me what life is like for a dog before being rescued? - Tell me about procedures of finding a right "match" between the dog and the owner? | **Notes:** |
| **Question 3:** What are the costs that are involved with rescuing dogs from abroad? For example, are there medical fees associated with importing dogs from certain geographical areas, or training fees for these dogs before they can be adopted? | |
| **Follow-ups:**   - How do you recuperate those costs? - Has there ever been a time where you couldn’t rescue a dog from abroad because it was too expensive? Why was that? | **Notes:** |
| **Question 4:** Tell me about some challenges you face through your work. | |
| **Follow-ups:**   - What can be done to reduce or eliminate these challenges? | **Notes:** |
| **Preamble:** *From what you’ve described during this interview, it must be very challenging and demanding to work in a rescue organization.*  **Question 5:** Please tell me what it is that keeps you continuing your work? | |
| **Follow-ups:**   - What do you enjoy the most in your work? - In the perfect world, what would the lives of all dogs look like for you? | **Notes:** |
| **Closing Question**  **Preamble:** *I’ve asked you a lot of questions today, and I want to thank you once again for sharing your thoughts and experiences with me.*  **Question 6:** Before I conclude this interview, I’m wondering if there’s anything I haven’t asked you about that you think it is important for me to know? | |
|  | **Notes:** |

**Conclusion**

- That concludes my interview. Thank you once again for making the time to participate in this interview and for sharing your knowledge and experience with me.
- The content of this interview will be transcribed (written out into a text) and used to highlight the perspectives and experiences of rescue organizations that import dogs to British Columbia, which is something missing from the scientific literature. However, your identity will be confidential, and will not be revealed at any point.
- Also, if you happen to know other rescue organizations that might also be interested in participating, would it be possible for you to send them my email or get me connected to them?
- Would you like to be notified once the report is available so that you can take a look at our findings?
- Thank you again, and I will be in touch with you soon!
- Turn off recording and end meeting
