# Supplementary material for: “It’s my calling”, Canadian dog rescuers’ motives and experiences for engaging in international dog rescue efforts
Source: PLoS One. 2024 May 31;19(5):e0300104. doi: 10.1371/journal.pone.0300104 (PMC11142615; doi:10.1371/journal.pone.0300104)
Supplement: S3 File — (DOCX) [file pone.0300104.s003.docx]

**Interview Transcripts**

**P1 Interview Transcript**

**Introductory question:** Can you please remind me, from which geographic areas does your rescue organization import dogs?

**Participant 1:** Alright, South Korea, Mexico, China, and Iran. Those are the primary ones at the moment.

**Question 1:** Can you tell me about how your organization came to the decision to rescue dogs from these geographical areas?

**Participant 1:** Yeah. Our founder, [Name of the founder], when she started this, she wanted to help dogs who had no hope in their country of origin, because people abuse them, neglect them, there’s no animal protection laws, or they have a problem with overpopulation. So, she targeted those countries where that was an issue.

**Follow-up:** So just to clarify, does that mean that your rescue organization was focused on foreign adoptions, and rescuing dogs from foreign countries?

**Participant 1:** Yes.

**Follow-up:** From previously talking to you, I remember that you have shelters or rescues that you work with in these countries. Can you please elaborate on how you got involved with these shelters in these source communities?

**Participant 1:** Um, well they actually reach… since I’ve been involved, it’s them reaching out to us. I don’t remember... I don’t know before I was involved - the original connection that was made, but since the years I’ve been involved, because we became known to international rescuers, they contact us and ask us for help. We have far more people contacting us asking if we can help them, than we are able to help. A lot of them have no other place they can send these dogs. They can’t get adopted in their country of origin, they’re just not interested, and it’s very hard for them to find anywhere that will accept them. So, they reach out to us and other organizations similar to us.

**Question 2:** Can you tell me about the factors you consider when selecting dogs to bring over from these overseas areas?

**Participant 1:** Yes, well we actually, before we agree to work with a partner rescue, we have a series of questions and requirements that they must meet, and part of that is their assessment of the dogs. So our assessment is basically part of that. Um, so behavioral assessment is important. They have to show us videos of the dog interacting with people, with other dogs, if the dog is suitable with cats and children, then we can promote that dog as being able to live with them but only if we’ve seen a video showing they can interact with a cat or a child. We have to have all health issues revealed to us. The dog does not have to be 100% whole, for example we often take special needs dogs but that has to be revealed; all information has to be provided on their health status. And… you know, we accept all sorts of dogs. We do not want to be... we’re not breed specific, we’re not... you know, age specific or any of that... we’re non-biased in that respect. We feel every dog deserves a chance, there’s a home pretty much for every dog, except for aggressive dogs. Which we can’t take because we’re not qualified to handle that.

**Follow-up:** So, for these assessments, sorry I might’ve missed what you said a little bit. So, these assessments are done by the shelters in the source communities?

**Participant 1:** Yes, we ask the shelters… We provide a list of what it is we expect if we are going to partner with these shelters. We ask them, you know, do you assess the dog to be friendly with people and can you verify that by means of a video. Strangers as well handling the dog. So uh, we can’t do it ourselves, because the dog is not with us, so we rely on them to conduct an assessment of the dog before we agree to post that dog on our website and make it available for adoption.

**Follow-up:** Can you describe to me what a life might be like before it gets adopted?

**Participant 1:** Uh, well, let’s take a dog on a meat farm in South Korea. There’s many of those that we found in wired cages, exposed to the elements, standing often in their own feces, there’s body of dogs around. We’ve heard this frequently. They’ve watched other dogs being slaughtered in horrible ways in front of their eyes. That’s a meat dog from Korea. There’s dogs in Iran, who. it’s illegal to keep a pet, a pet dog in Iran. If you have one you only take it out under the cover of darkness, because otherwise the dog would be taken from you and killed, and you can be imprisoned. So, dogs there, if they have the misfortune to be born in that country, they often are tortured by young kids on streets who think it’s fun to cut their ears and tails off. We have a lot of dogs from Iran who have been mutilated in that way. Umm, and you know they run them over, they’re considered ‘vermin’. So, that would be a dog in Iran. Mexico, it’s just a life on street. So, you know, risk of being run over, and starving to death. So, let’s see, China, they also eat a lot of dogs so they regularly round of up dogs, take them. There’s a meat festival, the Yulin meat festival, hundreds of thousands of dogs are killed and eaten. So, those are typical examples.

**Follow-up:** I remember when we previously talked, that you had some procedures to find the ‘right match’ between the dog and the owner. Can you please describe that to me again, what that was?

**Participant 1:** Well, we endeavor to post... To write a very detailed profile about the dog, so we get as much information as possible from the rescuer, we ask them to describe energy level, whether they bark a lot, whether they have separation anxiety, whether they get along with children, other dogs, these kind of things so we try and write a very detailed profile of the dog. We provide photographs and videos, and we put that on our website, and then we accept applications. So, people would see the dogs on the website, and they fill out a detailed application. So, we have a quite a lengthy application with a lot of questions. And our screening process then, it’s not a case of “that person wants that dog, okay, and the answer is yes”, we might get five applications for one dog, which especially happens for cute little dogs. So, we then have the volunteers who first of all, entirely read and screen the application with all questions. If it passes that stage then we have a phone interview, a different volunteer does a phone interview, we have a script that we ask them to follow and it sets a general direction, and if it passes that stage, then we either do a virtual home visit, we used to do in-person home visits but covid kind of put an end to that.

**Follow-up:** Right.

**Participant 1:** Either an actual home visit, or in some cases just photographs of the indoors and the outdoors of the house. So if they’ve gone through all of those stages, then that applicant is sent to, if the dog is from china, it would come to me, I am the rescue coordinator for China, we have different coordinators for different coordinators for different regions, and I determine which of those five applications for that dog, is the best fit, having all that information, and then the information about the dog.

**Follow-up:** Wow, that sounds like a lot of work. That’s incredible.

**Participant 1:** It’s a lot of work, that’s why we have to have a lot of volunteers because we’re all volunteers. It’s still not 100% fool proof. You know, sometimes it’s not the perfect match but it’s the best we can do.

**Question 3:** Thank you for sharing that. What are the costs that are involved with rescuing dogs from abroad?

**Participant 1:** Well, that’s sort of a very difficult issue, especially now. The way this works to best effect is when people are travelling a lot and they would agree to escort a dog. So, somebody happens to be flying, you know just a tourist or a businessperson, let’s say, flying from Shanghai to Vancouver which pre-Covid, happened all the time. And the rescuer in China and us, we’re always “promoting we need flight volunteers”, we call those people flight volunteers. um, there’s no cost to that person who agreed to take the dog in cargo, the dog goes in cargo, the rescuer pays the equivalent of excess luggage which used to be $100 to $200, the rescuer would pay that for the dog to be transported in cargo and come to Canada. So, for the rescuer that is their cost, plus the rescuer has to do the primary costs because they have all the vaccination costs, they have to have the dog health-checked before it leaves the vet, and they have to pay for a crate that is airline approved. Their costs typically would be more than the adoption fee we charge. So, let’s say they have a passenger, which doesn’t always happen, so $200 for that, the crate can be. in Iran the crate alone now is $500. Different countries. Typically $200 per crate, so that’s 400, all the vaccinations might be another couple of hundred, 600, and the microchip is minimal 25, there could be transport cost getting the dog from the shelter to the airport, so maybe $700, something like that. But of course they also bought all the food and the shelter, all that is costs as well.

Our costs on our end, are... We collect an adoption fee, which is typically around 700 and 85% of that is we send back to the rescuer to cover their cost. We keep like $100, $150 to apply to things like paying our fosters for dog food and things like that, paying our airport volunteers to go to the airport, we have to pay for their gas and their parking, we have vet bills for dogs that get returned and go into foster care, and we have the admin costs to keep have our website and that kind of thing.

**Follow-up:** Wow, that sounds very complicated. Has there ever been a time where you couldn’t rescue a dog from abroad because it was too expensive to rescue, or is that not really a case that happens?

**Participant 1:** Yes. Well, here’s an example of what’s happening right now. So I’m using China, because since covid nobody has been travelling. In fact, a lot of countries nobody is travelling but, China even now there is very few people travelling and nobody wants to be a flight volunteer it seems, so the only other way to get dogs here is to pay, have them flown cargo, which means no passenger. That costs $2,000 per dog. And that’s only because right now we have another organization who’s working to bring a cargo, a load of dogs, 26 dogs, it’s just dogs on this flight and that brings the cost down to $2,000 per dog, normally it would be $3,000 to $4,000. But this person has 26 dogs so they’re bringing it own to $2,000. So, $2,000 is way too much to ask for a rescue dog, you won’t get it. So, we have to fundraise, but we still collect the typical adoption fee, 750 or so, which goes very little towards that, doesn’t make a big dent in that $2,000, and the rest we have to try and fundraise for. We have not managed to cover the cost but the other organization, who is American, they’re coming up with most of it in this case. Typically though, that’s not an affordable option.

**Question 4:** Right. Can you tell me about some challenges you face through your work?

**Participant 1:** Yeah, well, that’s one, getting the dogs here is a huge challenge. Constant changes in the rules, customs rules is a big challenge. And getting enough volunteers to go to the airport which can take quite a lot of time, that’s a huge challenge. And then when it comes to the dogs themselves, we constantly emphasize through the adopters that when the dog first arrives they’re very stressed. It’s a stressful experience for a dogs to make that journey, and then to arrive in a place that’s, you know, smells, sounds, looks, completely different. Some of them have not lived in a home before, some of them are very afraid of people, so, as part of our screening, we’re looking for people who has that understanding that this is not going to be a North American dog who’s lived in a house, and is comfortable with all the same things. You need to understand that it will take the dog a little bit longer than other dogs to adapt.

**Follow-up:** So just to clarify that, one challenge would also be in the owner’s aspect as well, in finding the right owner who is understanding of the dog’s difficult backgrounds?

**Participant 1:** Yes, exactly. So that is something we look for, but it’s always hard to be sure. Some people will tell you all the right things and answer all the questions, and then when their dog is not reacting perfectly after the first week, they want to return it. Even though we said, it takes at least a month and, really before the dog’s true personality shows it could be 3 to 6 months and you need to be patient. Some people just can’t be, so, then and our commitment is always that we will rehome a dog or if it’s urgent we will take it away and pick a foster. So that is always a challenge because we don’t always have enough foster homes, it takes time to find another home that’s better, and that’s constant work in progress. But, let’s see what else. Those are kind of the main challenges. Oh, and there’s the emotional challenges, you get some horrible stories, and you begin to despair at people.

**Follow-up:** Is there anything that could be done to reduce or possibly even eliminate these challenges that you face?

**Participant 1:** Umm, well we’re always working on “rehomes” as we call them, you know, when the first adoptions didn’t work out. We’re always discussing how can we reduce that. Comparatively, it’s actually relatively a low number, for the ways to deal with that, tweaking the application process, can we make it a little bit more thorough, can we rule out people who say one thing and turn out to be different. And also can we make sure that dogs are as advertised. So, the information we provide is as accurate as possible, so we do that by developing more in-depth relationships with the rescuers. Other times, for example our South Korean rescue, we decided we wanted to introduce an additional video component where the dog is shown choosing to go toward a stranger, which is actually hugely challenging for a dog, for many dogs to do that.

**Follow-up:** And probably for those with difficult backgrounds especially.

**Participant 1:** Yes, so we can identify, this dog is actually going to choose to go towards a stranger that’s going to be an easier dog to place, he’s a bit more friendly than this dog who won’t go near strangers, and we’ll still place that dog but we’ll be able just to start that one as much more *inaudible* it will need, perhaps more experienced than this dog. So, constantly sort of looking at improving the information we get about the dog and improving from our end the process of screening the adopters.

**Preamble:** From what you’ve described during this interview, it must be very challenging and demanding to work in a rescue organization.

**Question 5:** Can you please tell me what it is that keeps you continuing your work?

**Participant 1:** Well, let’s see here’s one motivator *shows their dog*. He’s [my dog] from Bahrain. He was a dog that got me involved with this rescue organization. And hearing the stories from the adopters sometimes brings me to tears because they’re so moving, and so it feels often that you’re actually not only helping the dog, but you’re helping the person. And just seeing the transformation in the dog’s lives you know, from living on a dog meat farm and facing certain death to running along a beach in British Columbia and snuggling in bed with people, I mean the contrast is so huge and that’s really rewarding. And when you see the numbers of dog’s lives we have changed, dogs have a future that wouldn’t have otherwise had one.

**Follow-up:** That’s a very heartwarming answer. In the perfect world, what would the lives of all dogs look for you?

**Participant 1:** Umm, well they’d all have a home, shelter, food, and love. And you know, they’d all have a comfortable life. And they’d all be spayed and neutered so that this would not be a problem and there would not be breeders perpetuating number of dogs that can’t find a home. Everybody would adopt. Nobody would buy.

**Preamble:** I’ve asked you a lot of questions today, and I want to thank you once again for sharing your thoughts and experiences with me.

**Question 6:** Before I conclude this interview, I’m wondering if there’s anything I haven’t asked you about that you think it is important for me to know?

**Participant 1:** Well, there is a backlash, I think against international import of dogs. It’s not a very strong one at this point, but I worry that it might be growing. We’re kind of getting an indication of that from the States. I don’t know, have you heard that they recently introduced the ban on puppies?

**Follow-up:** The new law, yes.

**Participant 1:** And I worry that might cross into Canada. And you know we have vets that say “well, why import a dog from another country like that”, that say that to you. One of our adopters that brought their dog to be looked at or something. And we know there are local rescues, Canadian rescues that also don’t like what we do, so I think it would be helpful for your kind of study to show the positive side for example that, it’s not like we’re bringing dogs with a lot of diseases, that can spread in Canada, which some vets seem to think that. They’re all fully vaccinated and health-checked and the rest of it. And there’s a shortage of adoptable dogs in British Columbia. There is a need for what we do. It’s not like we are dumping British Columbia with unwanted dogs. We only bring them in when they have homes. So, are you looking at any of the negative attitudes towards international adoption?

**Follow-up:** Yea, so, exactly like you mentioned, I’m noticing that there’s two sides of these arguments. There’s one side that says there’s a shortage while there’s another side where they argue there is a shortage of dogs. So, through my research I’m trying to explore these conflicts.

**Participant 1:** Yeah, so we would certainly be happy to rescue dogs from the reservations in Canada where there isn’t so much spay and neuter, so they probably need homes. But we can’t get up there and we can’t get them here.

**Follow-up:** Right. Thank you for sharing that. I’m hoping that through my interview that I can explore these perspectives and get a bigger, fuller picture of what exactly is going with the rescue world.

**P2 Interview Transcript**

**Introductory question:** Can you please remind me, from which geographic areas does your rescue organization import dogs?

**Participant 2:** We generally get dogs from Mexico, specifically Puebla, Dominican, specifically Puerto Plata, Manitoba, specifically two reserves, [local reserve locations], Los Angeles California, Texas, an area in Texas and also in Tennessee, and then Egypt, umm I’d have to look up specifically where... Cayman Islands, Barbados, those are kind of the ones that come to mind, but I could maybe do a thoughtful chart later if I could think of any others. Oh! Costa Rica too! That’s big. Costa Rica, I missed that one. San Jose area.

**Question 1:** Can you tell me about how your organization came to the decision to rescue dogs from these geographic areas?

**Participant 2:** So, my sister who is the founder of [Rescue Organization], it would be awesome to interview as well, she was just working with another organization previous that kind of ended up fizzling out, so rescue partners were already kind of, we call them “rescue partners”, she knew of a couple, and then it kind of just trickled from there. I would honestly just say Facebook and social media in general has just really skyrocketed the ability to bring in dogs internationally. So, you know, one rescue partner talks to another and says, “hey how’d you ship dogs to Canada?” and they say “[Sister’s name]”, and then people were just messaging her on social.

**Follow-up:** Right, so, would you say then that your rescue organization was originally focused on international rescues?

**Participant 2:** Yes.

**Follow-up:** You mentioned that your rescue partners that you got in touch with, your sister already knew these rescues before starting off a rescue?

**Participant 2:** I feel like one or two? So the difference other organization she worked with was small dogs only whereas we mostly just take dogs that are needed to be taken, which a lot of the times are bigger dogs, but I think that rescue connections kind of just evolve, there’s a huge rescue community even in Toronto alone, so it’s easy to interconnect online through just talking dog, or for organizations to see what you’re doing from social media, and then… like I get reached out to every couple days on Instagram asking if were open to new rescue partners internationally, and asking how we can go about that process. So, I would say it probably helped to have had maybe like three connections through the other organization she was working with, and I think through that it was, here say talk chatter through the rescue community. I don’t know… I can understand that there might be a more pivotal desire to obtain specifics, and I mean she’d be a great person to interview to maybe get the true specifics of that.

**Follow-up:** Right. So once the rescue was established, you would just get contacted and it would just be... You wouldn’t have to reach out to any rescues?

**Participant 2:** Yeah, I would say we probably almost never had to reach out for relationships. We almost can’t support most connections that come forward anymore. So just for reference our one shelter in Mexico generally has about 300 dogs all the time, so if we take 20 dogs from them, they fill the 20-dog spot immediately so, it’s like a never-ending cycle. So, generally speaking we get reached out to by Mexico specifically a lot, but we just feel like it’s important to help with our one rescue partner that we’ve already established a relationship with and just continue to do the work there, but yeah it just snowballs, honestly. We get reached out from areas people that have heard of work that they’re doing to see if we can take adoptable dogs, because I think the main issue is that most rescue shelters internationally don’t do adoptions locally to their areas, so they need organizations like [Rescue Organization] to adopt their dogs out.

**Question 2:** From talking to other rescues, they mention that usually have some sort of factors they consider when selecting dogs to bring over. If you have any criteria that you for in dogs before selecting, can you tell me what these factors might be?

**Participant 2:** Yeah, so we help dogs locally as well and most dogs locally in Ontario have behavioral needs or medical needs, so when were able to take on dogs with medical and behavioural needs we typically try to support local cases. So when we’re looking for dogs internationally were hoping not to, I guess take over the place of a dog in need in Canada. So were looking for dogs that are more sociable with dogs, specifically, like good clean bill of health, unless there’s something specific that’s known that were kind of taking that on as a medical case specifically, but we do try to, the difference with Canada and… or I shouldn’t say Canada but Southern Ontario specifically and international rescue is that, international is typically an overpopulation problem whereas local rescue is typically more like, they’ve been failed through the system so it’s a very different type of rescue. So overpopulation problem we typically try to do our best to take in happy, excitable dogs that... I mean that’s like a poor way of explaining it but dogs that are “good dogs”, otherwise we have a lot of dogs here that, through the system that need more support. So I would say generally sociable, it can do well in an urban setting, so we don’t take dogs… like through the years we learned, street dog genetic is very important to understand and dogs that kind of, can thrive as stray dogs, and so it’s important that we don’t take dogs off the street that, like we take dogs that need human intervention. So, we kind of pick dogs that do well in urban settings, I guess.

**Follow-up:** Right, and that was a lot to uncover there and some of the concepts were pretty new to me. You mentioned that for local rescues, that you focus on dogs that the system failed them, can you elaborate on that a little bit?

**Participant 2:** So, yeah it’s a great point. That’s definitely an interpretation that I’ve created on my own through my experience. Most dogs in southern Ontario like, or I mean from my experience we don’t have a stray problem in southern Ontario. Northern Ontario has a stray problem. So, dogs that are typically in need of a rescue in southern Ontario have had homes that have not worked out for them. So usually, it’s a good point, I kind of claim that like “failed by the system” so, human education has not successfully helped these dogs to exist in the environment that they were bred or created for. So, that would mean that like someone wasn’t committed to the dog for its full life expectancy, and put it through the system, which would mean the shelter system, rescue system. Whereas the ideal is for a dog to be adopted or like you get a dog and you have that dog for its whole life. So like in other countries, generally dogs are stray dogs, so they’re being actually born on the street, they’re being born and thoughtfully... Just through procreation of dog to dog, strays, so they’re not created through, you know we buy dogs here in Canada for pets. They’re being created as almost like, wild species of animals these days. So, dogs here, they typically have distrust in something because they’ve been failed by humans before, whereas dogs in other countries they’ve been strays, and that is really important to understand their genetic build, because they are almost like on the verge of wild in some instances depending on how they’ve existed. Or some of them have also been owned and been through a system as well, a lot of them are abandoned as well, but I’d say it’s just like a different type of need

**Follow-up:** Thank you so much for elaborating on that. This is all really interesting. Can you describe to me what a “typical” dog might be for dogs that you rescue? It could be both for local dogs and for international dogs.

**Participant 2:** Sure. Yeah, so a typical international dog I would say would be a mutt. I would say it would probably has a hound... sighthound is a pretty typical dog, that’s like sleek, slender, maybe about 50 pounds, brown. There’s a lot of demonized terms for dogs but like “mongrel” is a term used for a type of dog in certain countries. But I compare it to like a squirrel here, because there’s so much inter-breeding they’ve created their own look. So it’s typically a medium everything, because it’s a mixture of dog, its like a medium-sized blend of every type of dog that exists. A more local dog I would say in Ontario, like we do Ontario, Quebec, and Manitoba rescue Northern, so a lot of those dogs are huge. They’re usually like 60 to 120 pounds, they’re usually a crossbreed between shepherd, husky, lab, collie maybe mix. Like each have their own particularities, the international dog typically are a little bit more cautious, fearful, apprehensive, and the local dogs are a little bit more confident, reactive on leash, more in-your-face, a little bit.

**Follow-up:** Can you describe to me what a life might be like before it gets adopted?

**Participant 2:** Yeah, I mean it’d be a great question for an international partner as well, because all I’m going to be able to do is generalize. But I think from our experiences, international dog probably born on the street from mom in generation of street dogs. Probably has to defend for itself, and or a litter of puppies could be born, the ones that survive either due to, whether it’s like, not having medical support or environmental concerns that come up, either they grow up on the street or they are picked up by a random civilian and then they’re kind of living in home environment which I would say is a little bit different than our context, like it’s more like an outdoor cat kind of space where you have freedom during the day, you co-exist with your community and then you go home at night or maybe have somewhere to eat. And then typically until something happens where human intervention is needed whether family doesn’t want the dog anymore, maybe the dog bit somebody, or is not fun anymore, maybe its barking at visitors or whatever, maybe the dog gets hit by a car, people can’t support its medical needs, or it doesn’t have access to food or water so its severely malnourished, maybe it has some sort of medical needs like skin conditions that are just making it unable to exist within society without some sort of concern. I would say that would be the point where someone would intervene and then bring that dog into a shelter.

And then I would say like our Northern dogs our kind of, also existing stray life as well, a lot of dogs are tied up there as well so a little bit different, and then we also in Canada or at least in these Northern areas have cold winters so they’re also having to live in these conditions which is different from what they’re experiencing generally in international spaces that we exist in. But one thing that happens up North is there’s dog calls, so a lot of times dogs are shot for being stray if they’re deemed either danger to society or there’s overpopulation, that’s kind of the way to solve things, so a lot of times we’ll be notified of those things through our rescue organizations, and they’ll ask us to help by going to do a pick-up of dogs. Or usually like right now like it’s almost turning to wintertime so would do what we could to get vulnerable dogs off of the street, so the dogs that probably wouldn’t make it through the winter months.

**Follow-up:** From talking to other rescue organizations, they’ve mentioned that they have some sort of procedure to find the ‘right match’ between the dog and the owner. If you have any procedure to find the right match between the dog and the adopter, can you please describe that to me?

**Participant 2:** Yeah we have like a big operation. I would say just the easiest way to explain it is we typically will bring our dogs into foster home and/or we work with trusted rescue organizations to figure out what the needs of that dog are, and then we look for adopters based off of building a bio and posting it. One thing that we’ve changed recently is we do trials, so every dog gets at least one to two weeks in the home that’s ideally going to be supporting them for their life to make sure it’s a good fit going forward. But our process is kind of like big news, so you can look more on our website if there’s any specifics, but it’s kind of an elaborate process that involves a lot of screening.

**Question 3:** What are the costs that are involved with rescuing dogs from abroad? For example, are there medical fees associated with importing dogs from certain geographical areas, or training fees for these dogs before they can be adopted?

**Participant 2:** Um, totally depends. Back in the day it could cost like $100 to bring in a dog where now, it’s a lot more expensive. I would say that our average cost per dog is about $1,300 because, it used to make sense to bring in dogs internationally until COVID hit and people weren’t travelling as much, so we started chartering planes for dogs which is just bananas. So, although the initial cost of rescuing the dog is done and paid for by our rescue partners, and then, when the dog comes to us, we pay for those expenses. So, we pay for the flight, which I would say is from 0 to 350 bucks let’s just say, some cases like from middle east could be $3,000 to ship a dog, so we only do those ones very infrequently. And then we also have to go through customs, and vet the dog so I would say that’s at least couple hundred bucks too. And then sometimes you learn that the dog needs surgery, like we just learned it costs $7,000 for that surgery from a dog from Costa Rica, or you learn that the dog has pet behavioral needs, it’s just not acclimatizing to the urban setting so it might need to go to training. So, I would say average dog let’s just say 1,200 bucks but it could be upwards of $10,000 depending on the case.

**Follow-up:** Would the main costs be medical costs, or would there be travel costs as well?

**Participant 2:** Yeah, travel costs are like always a thing. I would say travel costs 100% happen in rescue cost, but, let’s just say 250 per dog for travel, 250 per dog for vetting. And then, it could range. If you’re putting a range 250-500 for both intake and vetting. And then behavioral, you’re never looking less than 3 grand for behavioral thing, and then if a dog needs surgery then it’s like $3,000-$7,000 generally.

**Follow-up:** Has there ever been a time where you couldn’t rescue a dog from abroad because it was too expensive? Why was that?

**Participant 2:** Totally. Like, I think it’s something that were learning about. Like it’s just not feasible. Like we’ve had dogs that cost $10,000 and they still can’t acclimatize to their environment and it’s really difficult. I would say nowadays we definitely have to say no to dogs that are not going to vibe… like *Inaudible due to poor audio quality* flow in with what we have, I think it’s different if you have like a facility where you can house dogs but we have foster homes and people are only going to put up with so much. Again, we try to bring in dogs that are more particular. So I’d say it’s not like this “dog has issues”, sometimes we just can’t say yes to dogs that have a lot of unknowns, just because we don’t know if it’s something that we will be able to support. And I think it’s not always cost though, like I’m sure it’s an important question to ask. Sometimes it’s like “is this dog dangerous” or is this.. like, also just completely overdo it with our dogs in Canada like we almost over-vet them so it’s like a dog could have a limp and it’s like I don’t want it to have surgery but like people aren’t going to want it if it’s going to have a limp and they feel bad for it. So sometimes it’s just whether or not they can exist happily in this context.

**Question 4:** Can you tell me about some challenges you face through your work?

**Participant 2:** I think right now like just from what I’ve been writing about today… I mean what’s your focus of your thesis though? Maybe I could talk about specific challenges… I have so many challenges

**Follow-up:** I would like to know the main challenges that you encounter. Anything that comes to your mind would be your great.

**Participant 2:** But your focus is on international rescue right?

**Follow-up:** Um, yes. I guess in terms of rescuing dogs from abroad and importing them to Canada and as a worker in that context, what would be the main challenges that you face, which although as you have just mentioned you face a lot, but.

**Participant 2:** I think stigma is big. And you know, that’s stigma of the community, thinking what they think about rescue dogs. I think also like, it’s very rare that I meet someone at customs that I have a good rapport with. You know people in industry like customs and border security just don’t have our backs most of the time. And the laws around rescue, like you know it currently exists in a way that we can work and run most things, but it’s also really difficult. Like we can’t… without a puppy permit we can’t rescue dogs that are under 8 months and for me, like you want to get the dog as young as possible because you don’t want them to experience… like you want to climatize them as young as possible to make sure they have good development. So, if a dog is sitting in a shelter for 8 months just because there’s a law that it can’t come here earlier, I don’t see how that’s benefitting the welfare of the animal. So I have a huge problem with that. And it actually sucks because breeders don’t have... like the business of breeding dogs like its so much easier to bring in puppies from my experience. what else, umm…

I don’t know if it’s the answers you’re looking for but I think just kind of the fad of like rescuing dogs is difficult because like, it’s cool to rescue dogs, which is how we really became a big organization like don’t get me wrong we blew up on Instagram because were millennial organization which is not common for a lot of dog rescues that I know of but, it’s cool but are you committed to that and you know you’re taking a life of a living being and that comes with a lot of responsibility. I’m much more holistic in life like how I believe in things and I do believe in the idea of a living animal. And a lot of people see dogs as an accessory as a “pet” like I don’t like the term pet you know, your dog isn’t there to pet. I do this because I think it’s important. Umm what else… I think with Covid specifically, it was hard because as organizations that have learned how to in run a specific way for so long that we had to pivot, and it wasn’t fast enough for convenience of how we built our society. So you know, people were like “oh there’s no dogs”, like that was like a big conversation like “there’s no rescue dogs available” because everybody wanted a dog through covid and it was just that like we couldn’t access them in the same way like we are built off of the infrastructure of tourists so a lot people got Kijiji puppies which is really upsetting, and backyard breeders, like I’m not against breeding specifically I think there could be a lot of benefits for genetics of dogs that are needed for specific reasons but, a lot of unthoughtful decision making with dogs specifically. Is there anything that you’re like… If you could pull me in a direction of something I could just talk about random stuff forever.

**Follow-up:** No, this is perfect, and again, there’s no right or wrong answers. Anything that comes to your mind is perfect for me and that’s what I’m looking for. I don’t want to... kind of, guide you in a direction, but these answers that you’re providing are wonderful.

**Participant 2:** The one other thing is like, in just so many ways the stereotype or stigma of dogs from other countries bringing in diseases I think is like really harmful to our understanding of dog rescue. We rescued 2,400 dogs in, to be honest in a really unthoughtful way for a long time. Like, we didn’t know what we were doing from the get go, we just had a passion for dog rescue and we’ve learned all this through… you know, upped our protocol but like I cannot think of a time where a disease was passed on to an animal in Canada from one of our rescue dogs. And like I’m honest about that, although we have a handful of times brought in a dog that were sick and that’s really unfortunate, but I think that is one of the main reasons for the law of not bringing in puppies which I can also understand, it’s really easy for puppies to get sick or to have bad experiences on planes potentially, but I feel like that’s a really harmful way of understanding international rescue and demonizing an industry that is meant to support so many animals. I think there’s also like a lot of colonization in dog rescue and I’ve like learned that term recently. I think what’s important to us is like our rescue partners lead our interactions with them. You know we work on reserves up north and we you know, were there for them. We’re not trying to barge in with our expectations and things. I’ve been in many opportunities where I could buy dogs off people because I think they could live better in Toronto but that’s not what it’s about. It’s about supporting dogs where they currently exist. We do spay and neuter clinics in other countries and it’s hard to see dogs come in that aren’t living a standard of living that is similar to what I might see as beneficial, but I think it’s really about those small conversations or opportunities to share spaces with people and learn what they’re going through, like dog rescue is not the root of the problem. The way our society is built is the root of the problem and dogs are suffering as a consequence to everything else that everyone is experience. So, we just have to be sensitive to that in order to really imprint the space in a positive light I guess.

**Follow-up:** Is there anything that could be done to reduce or possibly even eliminate these challenges that you face?

**Participant 2:** Umm, it’s hard. So I run our social media, I don’t know if you’ve been on it but we have [a large number] followers on Instagram which is completely overwhelming and sometimes for me I truly believe like, I went to school for child needs studies, I believe in human rights, there’s a lot of intersectionality with dog rescue and there’s a time and a place where you want people to be followers and there’s also a time and place where you really want people to think critically and be curious. And you know sometimes we need people to be followers to donate and do the work that we need to be done so that we can be the experts and share wisdom and make thoughtful plans but I think like there’s just so much generational understandings of how dogs exist in spaces with humans that it’s a really delicate space, especially with… Wait what was your question again?

**Follow-up:** What can be done to reduce the challenges you face, but you going on these tangents is really interesting as well.

**Participant 2:** Uh, I think like, just being there for dogs in a manageable way. I think it’s such a simple thought but I’ve learned through so many years in an emotionally charged industry that I have to be there for myself in order to be there for dogs. And when I’m there for myself I’m there for dogs you know. I think we take on too much as a society and it’s like, people apply and they have like 5 kids and 3 cats and 2 dogs and I’m like “you don’t have any hands left for a dog” you know? I think the way that we run as a world is just like, of course were going to have dog problems. Like people reach out to me selfishly like they don’t want to help [Rescue Organization] they want to feel good about their involvement with rescue and hey I did that from the get-go when I started this place too like... I wasn’t trying to help dogs I was trying to feel less lonely after losing my first dog so, I get it, but it has really helped me appreciate charity work as well. I mean I also sometimes hate thinking about... like is the work that we’re doing just giving lax to the government for really having to own these responsibilities so I think that’s a hardship I face sometimes.

**Follow-up:** So from the things that you’ve mentioned, I’m getting a sense of feeling that, could it be a change in attitude from the collective, like everyone as a whole, would that be a critical factor in changing this?

**Participant 2:** Yeah, I think it’s just like… I love education but I hate education because I feel like the type of education that we offer and which is why I’m not in the world of child needs much is like… It’s like this or that. There’s the expert… there’s a teacher and there’s the student. Like were all human and I think we just need to learn how to be the extension of one another in order to make positive change. Because literally when I went to northern Manitoba which is the first time I had done rescue in a country where I spoke the same language, I’ve always ever been in like Spanish speaking countries, I realized in that moment that what I equated to that person that needed our help was that I was a plumber - I was coming in to do a service to fix the problem so you didn’t have to think about it ever again. And I think if we continue to think that everything else is everyone else’s problem and that like there’s these spaces that where we can just fix.... Oh the dog catcher! People would call me the “dog catcher”. They’re like “oh the dog catchers here, they’re gonna fix this problem that’s annoying, and were gonna get on with the rest of our lives” it’s like, everything’s intersected so you can just put pockets of things… Like compartmentalize it all.

**Preamble:** From what you’ve described during this interview, it must be very challenging and demanding to work in a rescue organization.

**Question 5:** Can you please tell me what it is that keeps you continuing your work?

**Participant 2:** Hmm. Yeah, I think about that sometimes. I think for me like I’m in it for the community, less about the dog specific, which I don’t think is known to a lot of people but, like dog rescues a foot in the door to so many ways to benefit or change and support the world. I think like whatever the focus is that were each doing, we’re all intersecting so you know, I can appreciate that I can have the experience and time to focus on this one thing that is dogs, and then I also, you know expect and hope that I can relate and call on other experts and community members that can help lead in the areas we intersect with them and the areas they intersect with us. So, I really do think it’s a collaboration always. Dogs also teach… like I learned everything first through dogs like I, most of the work I learned is first on a dog walk with my own dog and I learned it about humans, it’s like “oh that actually really relates to humans” and then it relates to communities as well so, um… I don’t know if that answers what I was being asked but…

**Follow-up:** Yes, this is great. By “community”, who are you referring to? Can you elaborate on that?

**Participant 2:** Umm, I guess just the people around us… Can you remind me what exact sentence that made you ask that?

**Follow-up:** So you mentioned that you were in “it for the community”, so would it be the community you are rescuing the dogs from, or would it be more in local?

**Participant 2:** I think for me it’s almost like… I mean this isn’t what I was really saying but I guess this is like a different thought about community is, you know creating an organization from the ground up you really get to practice your own community, in a way that has value systems that align with what you believe you should see in the world so, probably I’m a very controlling person in a very passive way. So, it felt a little bit like an experiment to be honest, like, “can this work”.

**Follow-up:** That’s a very heartwarming answer. So, in the perfect world, what would the lives of all dogs look for you?

**Participant 2:** Oh. I think that dogs, human, nature, communities could just co-exist and that we would be mindful of one another and not necessarily need control one other’s spaces but allow for each other to build happiness and you know, evolve one another instead of harm. I would love to live in a world where dogs don’t need to be leashed. I think that that’s such an Americanized way of supporting dogs. Or that we have backyard spaces, we really just like to control and own things and dogs are property. Which, I don’t really know how to move away from that but I think that, you know, just like I might feed the bumble bees in my backyard by planting plants, you know, I think there’s a way to do things that’s a little bit more respectful, but harmonistic. So, I think that there’d be more like a blend, and less of like a definitive “you own this dog, you’re 100% responsible”. I also think it’d be grand if we didn’t have so many dogs on the planet. Because I think it’s really unsustainable. I really like community development in that like you know, a lot of people are having a time caring for their animals and it’s like, what if we just like… I live in a court, like what if we just had a court dog. Why do we need everyone to have their own dog, and like everyone has to walk, feed, and clean after their dog every single day? What if we shared those responsibilities so that its manageable? So that we don’t have our hands always preoccupied, and we could focus on ourselves, and our family, and our community altogether. Instead of like, I feel like a lot of things are individualized. If someone wanted to walk my dog for me once a week, that would be so lovely and I would want to naturally give back to them just for their involvement in supporting me and a lot of people really want to be around dogs. So sometimes I’m confused of like, we want to be around dogs, but we don’t help each other’s dogs out, we just want to own our own dogs.

**Preamble:** I’ve asked you a lot of questions today, and I want to thank you once again for sharing your thoughts and experiences with me.

**Question 6:** Before I conclude this interview, I’m wondering if there’s anything I haven’t asked you about that you think it is important for me to know?

**Participant 2:** Can I ask you a question first?

**Follow-up:** Of course!

**Participant 2:** What brought you to this space that you’re in so I can just understand of your direction?

**Follow-up:** So, why I am doing these interviews?

**Participant 2:** Why are you doing animal welfare in school?

**Follow-up:** Oh, haha. Um, growing up in Tokyo Japan, even though I didn’t have a lot of interactions with animals, I always really loved being around animals and I was always petting stray cats there, and, every summer I would visit farms in Germany and interact with animals so I knew I loved animals and just being around them. But I didn’t even know the concept of animal welfare growing up at the time in Japan and I just wanted to learn more. And so, for university I came to UBC, into the program that I am still in, now for my Master’s. But at the time as an undergrad, and that’s when I first heard of animal welfare, and the science for helping these animals and that really resonated with me and I just really loved the program and that’s kind of why I stuck throughout and I’m doing my Master’s in the program. But yeah, that’s kind of my position in this research and why I’m doing the current work I am.

**Participant 2:** And did someone start this research and you just jumped into it with them? Or like did you think of this thesis idea from the ground up?

**Follow-up:** Right. So it was my supervisor, my current supervisor that suggested the idea about dog importation and how it was growing but there wasn’t a lot of research done. And when I first started, I didn’t know anything about it, but as I did my research and learned more about it, I just got increasingly interested, and now I’m doing this interview to figure out my own questions that I had on, why is it that there’s this conflict on… that there’s people saying “oh, don’t bring in dogs” but then there’s a lot of rescue organizations that are actively bring in dogs so I wanted to understand that conflict, and so.. yeah, that’s what brings me to these interviews.

**Participant 2:** Yeah… Interesting. Yeah, I think that it’s interesting that there’s conflict of opinions, with everything like... We just so openly share how we feel about things without even having to lift a finger a lot of times or be accountable to the actions we put forward. Like I get tagged all the time in dogs that are on euthanasia lists and, I think in my head I’m like “are you willing to do anything or are you just passing off this information because I have to now take on this responsibility and you feel good about it”. It’s really easy to turn against a lot of people because it’s such an industry that just sucks you dry, which I’m sure a lot of charities find. But I do think it’s really important even through the conflict of everything to listen. I think that’s really important and again, I’m here to grow just as much that anyone that comes through our organization is growing as well. When I do an adoption interview I’m learning just as much as they’re learning. It might not be exactly the same type of learning but I think is just really important to stay open minded, and to… Like stay open minded with what people like to bring forward to me and also stay open minded.. I don’t know in every which way but… yeah, because it’s really easy to play the victim and be overwhelmed and not have to pass any time to listen to why my supporters are here and what’s happening. I think too that it’s also important not to just like, give into what other people are suggesting that you do. Like I think I’ve just thought for so long that as an organization we had to abide by what people are complaining about and I think I’ve just realized just like a business, if you don’t really believe in the value system then you can absolutely support something different. A lot of people don’t like international rescue and that’s okay, you don’t have to support an organization but I think you also don’t have the right to just tell us that we need to change and be different. Because a lot of people just give suggestions like that and it’s kind of mind boggling. Like there’s a lot of benefit to international and there’s a lot of benefits to local rescue too. But I think we all deserve to live all together a little bit.

**Follow-up:** And just to clarify a little bit, your rescue does both local and international.

**Participant 2:** It does both. I would say we’re predominantly international, and I think the reason behind that which is very interesting, it’s because it’s easy. It’s so easy to… and I mean that is laughable because I would need to drink alcohol to say that, because it’s not. What’s easy is that, dogs are accessible and in need in other countries, whereas its actually... like building relationships with local people and doing so in a way that’s really sensitive and beautiful is a lot more difficult for some reason locally. Whereas internationally again, all these dogs are in abundance you know, there’s so much variety whereas, local work is… It’s hard. And also the costs are so different. You work with a dog in Dominican, like it costs me 30 bucks to neuter a dog, like it costs me $500 to neuter a dog here. The Americanization of dog welfare… Well actually I wouldn’t even say dog welfare; the pet industry is whack. So, you’re dealing with the consequences of that when you deal with local work.

**Follow-up:** Right, thank you so much for these full responses. So, I think that’s all for the interview, so I will now conclude the interview.

**P3 Interview Transcript**

**Introductory question:** Can you please remind me, from which geographic areas does your rescue organization import dogs?

**Participant 3:** Specifically outside of Canada?

**Follow-up:** Yes, I would be more interested in outside of Canada.

**Participant 3:** Yeah, outside of Canada, oh man. Well, that constantly changes because we are adding on new rescue organizations that we work with in new countries but for right now, the areas which we have imported or are continuing to importing from would be California, so united states, Taiwan, Mexico, Lebanon, possibly Afghanistan, were trying to help some of the dogs get out with what’s happening over there since there is a ban right now in the United States from 112 countries with imports coming in. Their only option is to obviously send them to be housed here in Canada so Afghanistan could be added to the list, I’ve helped dogs out of Romania before, um, oh man I’m trying to think. Saudi Arabia had dogs flown in once. Um, but those are probably the general countries that we have helped from with some of those being continuous as to this point.

**Question 1:** Can you tell me about how your organization came to the decision to rescue dogs from these areas?

**Participant 3:** Well, I mean in general we’ve always wanted to help outside of Canada as well because we don’t really believe in man-made borders when it comes to helping animals in that aspect, or humans to be quite frank with you. But each their own on that. Um, so for us we’ve always kept an open mind in kind of a spot on rescue that helps dogs from around the world that literally have no chance and because we do have usually have such a great demographic of people here who do want dogs, it tends to help. Now, I guess each would vary, it depends on... we’ve met people through just friends. Friend’s rescues who have friends in other countries that need help so we’ve met them through there. Whether they’ve reached to us through private email, some of them have, or calls as well. Yeah I’d say usually we probably somehow connected with these people either through other rescue friends of ours or them directly reaching out to us and we just researching them and the work they’ve done and, you know what people tends to have experience with them, and if they seem credible, were open to helping. Especially if they are from a country where it’s very difficult for animals and for people in general. So, we try to take those ones as top precedented as that tends to be where most help is needed. Yeah, I would say that’s certainly it, just through friends or private contacts.

**Follow-up:** Was your rescue organization was focused on foreign adoptions, and rescuing dogs from foreign countries?

**Participant 3:** When we first started [Rescue Organization] we actually probably did a bit more international than local. But then we started to kind of do 50-50, and then we went, because we naturally grew, it’s not like we stayed the same size and decided to cut out import a lot more than domestic, it’s that we started to grow so we actually opened up a lot more to being able to help here. So, as we grew we actually ended up doing more domestically in Canada and in BC, and I wouldn’t say less import, we just, as we grew, we grew bigger within Canada and continued to do our import that we were already doing with increasing that slightly, but I would say since we started, it was probably more import than where we are now, is more domestic, but that doesn’t mean we still don’t help with as much import, it’s just because we’ve grown so much since the first day 7 years ago to now, including with all the program and outreach we do here in BC and Canada, so that’s one of those reasons. It wasn’t in regards to we wanted to slow down or stop doing import it was just the way it naturally grew that way. But we are always still trying to help more placed for import, each day to be honest with you, especially these days with what’s going on in the world.

**Follow-up:** So, when you first started your organization, can you elaborate on how you got involved with these fosters or other groups that you might have in these geographical areas?

**Participant 3:** Yeah, sure, so I was already raised around animals my whole life through my mother, she helped protect animals for the government for over 3 decades, so I was always surrounded by animals in my life and taught how to be compassionate towards them and then, into my early 20s I got into a rescue with another organization, I actually started from some trips to Arizona when it really opened up my eyes on what’s going on in the world and there, due to overpopulation in dogs and shelters and then, from there I joined a group, another rescue and I was just a volunteer at first and then overtime I ended up being on the board of directors, and then that group kind of disbanded years later and I just felt like this was part of what I wanted to do with the rest of my life, like it was my calling. I just felt so passionate about it that when they disbanded I was like “this can’t be it”. So, as somebody who has always been kind of an entrepreneur myself anyways, I was like “why don’t I start my own?”. And at the time I had a partner that was like “yeah, let’s do that!”. So, we just rolled right into leaving that group as they disbanded and just rolled right into starting our own. And here we are 7 years later. So, that’s kind of how it happened. Just through the transition of that one shutting down and not wanting to stop the work we were doing and, always a love for kind of being my own boss and starting something so, I just went and took the leap of faith and here we are.

**Follow-up:** Right. And I’m sure that now that your rescue organization is widely known, you get a lot of messages from other groups asking for help. But when you first started your rescue organization, was that the case or did you have to reach out to other rescues to bring in dogs?

**Participant 3:** Yeah, definitely when you first start, especially back then, it was really hard. When you start a rescue sometimes, at least back in the day, you were more criticized by the rescues than anything. You didn’t really have people reaching out to you, wanting to work with you or to help with as far as other rescues go. People needing help with personally with their dogs, yes, as they get to hear more about you. But no, when we started we only had a few connections to a few places so we just kept taking places from there, and so we branched out but definitely when you start in this rescue world or at least when you did when I started, it was actually pretty brutal. You were really criticized by other rescues, and they actually tried to… yeah it wasn’t very supportive unfortunately. Now, I think times are changing, younger people are getting involved, we’re seeing that we can do better work together as one and a part, but I think like any business there’s always people who just refuse to work with others. They just...They don’t… I don’t know, they don’t match up. Maybe with their rescue ethics and morals I’m not sure. But no, we didn’t have anyone reaching out to us at first, ha ha, absolutely not. We had to make our own connections and reach out to some people we already knew through the previous group that I was a part of and continue to use some of those connections to help. So no it took over 3 years, 2 and a half to 3 years until we really solidified our name, got the respect we deserved and the credibility, and had people stop to, basically criticize us to a certain extent. I mean people always do that to rescues and all companies and people but, in the beginning like, holy man it was really tough. Yeah, we were victimized quite a bit actually it’s sad. But times have changed, so no, sorry, ha ha, the simple answer to your question is, no we were not reached out to by other groups to help in the beginning at all. It wasn’t until we built our own name, our brand, and reputation, that they started to do it. And then as I connected in with more groups and became part of the [International Organization] as well, that’s when it really pours out. As soon as another international group posts about how much you have helped them, it spreads. I mean you get messages from Korea, China, India, Pakistan, Mexico, Brazil, you know, they all just start coming out of the woodwork because it’s hard to send dogs that far I think, and truly trust people unless… and most of these groups will never get the chance to meet you in person, so I think yeah, anyways that’s the big long answer to the very simple question.

**Follow-up:** No, that’s wonderful, thank you so much for the complete answer. So, talking to other rescues, they mention that they have factors they consider when selecting dogs to dogs over. Does your rescue also have factors you consider, and if so, can you tell me about them?

**Participant 3:** Well, I think any factor of taking on any dog whether it’s domestically or internationally is temperament. What’s going on with the dog, what is this dog’s needs, you know. Is it just scared of people or is it actually trying to bite people? Is it scared of dogs or is it trying to bite dogs? Is it great with dogs? Great with people? Or just not used to a city environment, or just not used to being on leash, or say in a house. So there’s many factors that come into play. I think number one in importing is health. So, has this dog been tested for rabies, or for a tick-borne disease, or for bacterial infections, or for something that you know, we do or do not have here. But regardless we don’t want a dog to be sick when flying and we don’t want to necessarily bring in any sickness into this country. Especially one that’s not in this country already. That’s very bad, right. You never want to have that happen. So, you want to do your due diligence in making sure that you ask these rescues “I want to see all these medical reports. I want to see them very current and up to date. And if they’re not or you can’t afford it, we’ll pay for it. Go get this bloodwork done and show me the results. Go get this SNAP test done and show me the results.” To try to really make sure. And really it’s ultimately to make sure the dog’s healthy, right? But obviously we don’t want to bring in a sick dog especially on a plane. That’s never good for anyone. So, I think health is number one, which will roll into temperament as well. Now, we’re pretty open here because we have a lot of experience with rehabilitating dogs with all sorts of behavioral issues. Some rescues pick and choose very wisely where they are like “We just can’t take on a dog like this, I’m sorry it’s too much for us” or like “Oh we only take small breeds we can’t take these bigger dogs”. So, a lot of rescues will have their specifics understandably, and that doesn’t make us any better but we try to stay pretty open. Where I’m like “You have a Great Dane? We’ll take it. You got a 17-year-old chihuahua? We’ll take it.” you know what I mean? We’re pretty open to helping all dogs, but yeah of course if a dog shows up here and is literally trying to attack humans, that’s a serious problem. And I don’t just mean like, it’s scared and I cornered it to pet it at someone’s house and it nipped me. I mean like it saw a human, and like went, that’s bad news right? But most rescues to be honest, would never send a dog like that over. But even if they didn’t see it then, sometimes these temperaments don’t come out until they get here. So, a dog living in a shelter in Morocco… Oh, there’s another country we actually help in – Morocco, my friend runs a rescue there. So, sometimes dogs can be in a shelter with a hundred dogs there acting perfectly fine, but then when you bring them here, they’re super reactive. But that’s also because you’re forced to act proper when you’re put into a shelter like that, because they know that if they start trouble, there’s going to be a hundred dogs going after them. So they tend to act right really quickly, because they’re forced to. It’s kind of like putting a bully into a room with a bunch of bigger guys they’ve never met, you know, they generally aren’t a bully anymore. Do you know what I mean? So, temperaments can change, but generally number one is health. Whenever you’re importing any animal into another country, health has got to be number one. You’ve got to realize what’s already going on, what the dogs had or continues to have, especially with heartworm too and stuff like that. But obviously you can treat all these things. And it’s usually cheaper in those countries to treat them. So if they are really sick, we’re like “keep them there”, obviously. “Never fly a sick dog”, but at the same time, we’ll treat them. Like we’ll gladly help donate that money because it’s probably a tenth of the cost to treat there than here. So, do everything there as much as you can and check for temperament before you bring them. That’s the biggest thing for us. Really just health and temperament, and the credibility of the rescue.

**Follow-up:** I know that you described how the dogs that you bring in, you are quite generalistic with the breed and you are not specific so this question might be a bit of a challenge, but can you describe to me what a “typical” dog might be that you rescue?

**Participant 3:** Probably on average a mixed-breed. So, not a purebred. But there’s tons of purebreds in rescues and shelters so we do get them. But on average, the common dog as you ask is probably a mixed breed. Probably an adult ranging from between 2-5 years old. And they’re probably somewhere between 40 to 65 pounds. Mixed-breed 40 to 65 pounds is generally your common rescue dog. But, I mean any dog can be a rescue dog. I’ve had a purebred German shepherd, purebred Frenchies (French bulldogs). When you think about around the world, street dogs, around rural communities, most of them are just these adult mutt dogs that were just either born there or dumped there at some point. But, rescue dog can be any type of dog. In California you can walk into shelters and it’s like the Costco of dogs. You could find Frenchies, Boston, pugs, German shepherds, huskies, malamutes, Belgian Malinois, I mean it’s crazy. So, the fact that people say “you can’t find them unless you go to a breeder. Haha, I could find them for you in a heartbeat, you just need to know where to look”. But the common dogs for rescues are medium to large sized mutt. We get an eclectic mix. We also house a lot of puppies because we work with a lot of rural communities and with indigenous communities. So, a lot of puppies are unwillingly born there and people just want to help them but they don’t have many resources. So, it’s hard when you live in the middle of nowhere so we tend to reach out there so we get a lot of puppies here at [Rescue Organization]. It’s because they’re vulnerable. We focus on all dogs but when it comes to puppies or really old seniors well take those in a heartbeat because no senior should live a life at a shelter or go down because they’re old, and puppies have their whole life ahead of them. But again, we each dogs seriously regardless of age. I think the more vulnerable are the ones you’re trying to get out as soon as possible because they’re at higher risk of getting sick or dying.

**Follow-up:** Got it. And This again might be a bit challenging to answer because you work with such a various spectrum of rescues, but what is life like for a dog before being rescued?

**Participant 3:** I guess it depends on the situation they’re rescued from. I could get a dog right from your next-door neighbour that’s just never been trained. That’s all over the place. I could get a dog that’s been literally living in a kennel for half of life or chained up for most of its life, being neglected, beaten, so that’s a difficult question to answer as there is a big variance in those aspects. But what has a basic rescue dog been through, probably some form of neglect or abuse. Lack of attention, lack of care, lack of love. That’s probably your general rescue dog. But that’s a very diverse question because what is considered a “rescue”. So, I think that could vary in so many ways. So generally, a dog that’s not a local surrender that were getting has generally been abused, neglected, or mistreated. But, you know, there’s all sorts of spectrum on rescue dog from your next-door neighbor, to out of a shelter, to finding them chained up you know, half dead. It does vary. Sorry to be graphic, but um. Yeah, I hope that answers your question. But most of them has gone through some sort of trauma.

**Follow-up:** From talking to other rescues as well, they often have some sort of procedure of finding the right match between the dog and the owner, like a “right fit”. I was wondering if your rescue also has that and if you do, do you mind describing that?

**Participant 3:** Sure. So each rescue has their own processes on how to do them. Ours isn’t specific to us, other people use this process but each rescue can tweak their own. So for us, we do a foster to adopt trials. So basically, we post a dog up for adoption on our platforms, websites, Facebook, Instagram, social media. I will post a dog and people will apply to our website. Once they apply on the website, and of course they can message us questions but hopefully the bio will answer majority of the questions they want to answer. We get people messaging people messaging, people replying on our website, we’ll then print out those messages and review them, kind of like how people would review a test in school. We’ll grade them, put little notes on the side, and then we’ll look them all over and look and give them a grade like A to C. Whatever they are we’ll find the top best fit. And the one they’re rated a C, it doesn’t mean that they’re not suitable for adoption, I’m just talking particular for this dog. So if someone’s like “I want a border collie that’s active”, but they’re like “but I’m lazy”, ha ha obviously they might get a C in comparison to if they wanted a chihuahua or an older dog they might get an... So, we’ll basically review their application, we’ll call them on the phone, ask them all the questions we need to ask, we’ll ask them to ask us any questions that they have, if we still feel comfortable after that interview, we’ll do the home check. So, some form of way to look what their home looks like the way they look, and then if all looks suitable we’ll meet the dog, go for a walk, a chat, maybe have the dog go out with some of our dogs. We try to show them the different aspects of the dog. And after the meeting they’ll usually go home, usually stay for the night to let it resonate, and then by next day let us know what you think. And if we both agree that it’s a good fit, then we can start what’s called a “foster to adopt”. This is up to a 3-week trial where the dog will come into their home, we supply them with all the supplies they’ll need at 24-hour support, and we just take it day to day as a team, and work through anything that come up. The ups and downs, the questions that they have, the things that we’ll need to come and work through and I think within that 3 weeks, us and that particular person will start to understand whether they think that dog’s well suited fit for them moving forward, even if it still needs training or work, which most dogs do especially in a new home. Or, whether we don’t think it’s a good fit, and the dog simply comes back to us with all of the items and we try again with those people to try and find the right fit. Obviously we are trying to screen, so we generally find that right fit the first time, it can take two times, rarely three times and that’s okay. Yeah, I’d say that’s generally it. And everything goes really well, we’ll go over there and finalize with official an official contract, make sure they have the original medical records, and we’re always here for them. So whether they message us 6 days later, 6 weeks, 6 years were always here for that dog to support them for any questions for anything that comes up. For the life of a dog it always has to come back to [Rescue Organization]. So if you adopt a dog and 14 years later you have to give it up, it has to come back to us. We just don’t want our dogs thrown into shelters or online, bounced around. So that’s kind of our oath to the dogs as well as the people, that we’ll always take them back. So, yeah that’s kind of the best way to do it. We just feel it out, it’s a no pressure process, and we just hope for the best. Yeah, we only specifically pick people we feel are suitable. Obviously if they have kids, or dogs, or other cats, those are more variables so we might have to do two or three meetings, we might have to go into their home more and have more interactions just to make sure. If someone’s like “Oh it’s just me and my partner and one works from home and we have a house and a backyard and we run”, it’s just like, almost any dog can fit into it, right? But if somebody is like “Oh we have three kids, two dogs, a cat, and a horse and chickens outside and we need a dog” I’m like oh lord… So it’s not like we can’t make that work, but obviously that’s going to be a lot more time consuming, you have to be a lot more careful, safety is number one. But for families with kids and dogs, there’s the variable from kids, and so are dogs with kids because they make a lot of noise, they move fast, scream, can be very abrupt. Some dogs are just naturally okay with it but, I’d say that’s generally the process. We just try to do the best as we can with being thorough as we can and support is everything. Just support for these people is everything so I think that’s very important.

**Question 3:** Thank you for elaborating on that in such great detail. This next question might be a bit more sensitive so I understand if you do not want to answer this, but what are the costs that are involved with rescuing dogs from abroad?

**Participant 3:** So, sometimes the rescues in those other countries have fundraised and paid for most of their cost, sometimes they don’t, sometimes you split them, sometimes you just send them back a fraction of the adoption fee, I guess it depends on the agreement per independent rescue. But generally the cost would obviously be medical to start. And from medical to care, and there to flights. So, flying from whatever country to here. If they have someone who goes on the plane with the dog who’s already coming to this country, so much cheaper. If they’re just trying to fly with cargo with nobody on, one, way more stressful for the dog, two, it’s like five times the cost... It’s crazy. So I would say initially it’s just obviously the health care, the vetting, and then the food, then it would be the plane, then from the plane they arrive here, we might have to follow up with vetting. The biggest cost for importing animals is generally vetting and transport. And that’s like all around the world. If I just go down the road and pick up a dog, still the most expensive part of that is generally their vet cost. But if you look at time and effort put in into monetary value, that might be. But just in general, like you’re asking, what’s the initial monetary cost, those are. But if you took in the time and care it takes for some dogs to be in rehab and put that into monetary value, oh that would probably blow everything out of the water. You know, because some dogs take six months to be rehabilitated and that’s a lot of time and work, especially if they’re not good with other dogs or people. The amount of care, time, and effort you have to put in to keep everyone safe, that’s a lot. So, yeah, transport and vetting is always the most expensive. And if you want to put other levels into that monetary value, care. Ultimate care. Rehabilitation. Even with rehabilitation, whether either you’re going to hydrotherapy, laser therapy, physiotherapy, whether you’re just doing socialization with other dogs, it’s expensive, and definitely time consuming. But, that’s why you’ve got to love it. You’ve got to love it because it doesn’t pay you.

**Follow-up:** You might’ve already touched on this slightly, but how do you recuperate these costs?

**Participant 3:** Well, basically adoption fees. Sometimes you get money back through your adoption fees, so we try to cycle those. So, if we rescue a dog that’s needing basic spay and neuter and vaccine and some socialization, generally we can recuperate within the adoption fee. We try to get discounts from our vets try to fit those within, but there’s a lot of times you never do, and that’s rescue. I mean, to actually make money within rescue, to have a profit in rescue to either pay employees or to have a buffer zone, it’s really difficult. But it also depends on the rescue. If you just take on so many cases, you’re just flowing through like we are, but if you took very specific ones and set the worst cases for fundraising, you could probably create a big buck from rescue. We do a lot of outreach and other programs where we use money like… it just comes and goes. No one gets paid at [our Rescue Organization]. No one. We are all volunteers. I’ve been a volunteer for 7 years in my own rescue. So fundraising events, events at pet stores, whether we’re posting online on our platforms, I would say that’s probably the biggest way. Then I would say probably fundraisers for whether you’re selling auctions, doing them at pet stores, or whether you have a tattoo event, those are great. Or some sort of event might happen. Maybe a barber shop does an event or, you never know. So, fundraising is a big way to offset those costs. You have to be better at fundraising in order to become bigger because you will not be able to offset those costs with simple adoption fees, no way. So, I could have a dog that I adopt out, a puppy, let’s say for $600, and it could’ve costed me $3,000. I would never get the money back unless I posted it pre-hand or recouped it in that sense. So, yeah, fundraising, recycling adoption fees, and rarely you will get corporate sponsorships but that’s just for that month, or one time, or monthly or yearly, and then you can start putting on like pension lists for people. So different government organizations have like charities that they could put on lists, and people that work within those companies can choose like $10 to $15 a month to that charity and just leave it. So, if you get big enough and if you know people, getting in those spots is great because its constant funds coming in each month without even thinking about it, because it’s just coming out of their bank; you don’t have to fundraise or advertise about it. That’s a great way to get going. We’re just getting our foot in the door there and it’s been 7 years so, that’s probably my rough breakdown.

**Follow-ups:** Has there ever been a time where you couldn’t rescue a dog from abroad because it was too expensive? Why was that?

**Participant 3:** There’s only one dog that pops into my mind that’s in Lebanon at my friends rescue at beta, and he’s a Caucasian shepherd and he’s got 230 pounds, he’s huge. I want rescue him so badly but we have not been able to find an airliner that’s able to take him and if we hire a private charter plane it could be like $75,000 to $150,000 so probably that. I wish I could bring him on a normal plane I’d even pay 5-grand for different styles of crates to get one made but we haven’t had any luck. Especially since its Lebanon too, so it’s very far for a dog, we can fly dogs from Lebanon but a 230-pound dog, to be lifted up out people, to be put in and out, and a Caucasian shepherd, getting that dog out of its kennel… So, I would say that’s the only one that comes to mind. I’m sure there’s rescues all around the world and possibly us at one point where were like “oh man, we don’t have that $3,000, $4,000 it would cost to fly that dog from India right now”. So, I would say yes, that is a factor in a lot of rescues in general. But I can’t think of a specific case besides that dog, where we haven’t been able to somehow make it work in the long run. But rescues everyday struggle with finances to get dogs imported in or out or just in general so. Yeah I’d there has definitely a few cases out there that we’ve had but we’ve been able to accomplish them, but the one that’s outstanding to date that I have not been able to is that dog. Yeah that dog I really want so, I’ll just continue to get that dog out whatever I can’t but that’s a mission in it of itself.

**Follow-up:** I could imagine. I’d love to hear an update when you manage to do that somehow.

**Participant 3:** Yeah, I’ll let you know. It’s a rad dog. It’s actually on my Instagram or Facebook. There’s a photo of me with a huge dog with his tongue out, it’s huge, that’s him. So, yeah I’d say that’s the only case, but that’s a common thing too. Not being able to import due to costs and flight embargos too. Certain countries won’t fly during certain times of the year because of the heat. And especially if it’s stub-nosed dogs like Frenchies… Uhh, yeah, so that’s basically it.

**Question 4:** Tell me about some challenges you face through your work.

**Participant 3:** Oh man… I mean, obviously there’s lots of challenges… Finances. It’s huge. It’s probably the most for any rescue. If we were all millionaires, the world would be a different place, right? If we had endless funding, my god. We’re independent people basically taking care of a global government issue. Each countries’ overpopulation is their issue, right? But they don’t address it so we do. So be it. So I’d say finances is probably the biggest struggle through and through for any rescue. Support, as far as volunteers or employees, especially ones that really stick around because it can really defeat you this type of work. It can make the most positive person that wants to help animals become… yeah... it’s really hard. PTSD is a huge reality. A lot of people, SPCA (Society of Prevention to Cruelty to Animals), cruelty officers and others have to quit due to that, and I’ve experienced it myself many times and I think even the strongest of minds break. I won’t lie if I say I haven’t broken down a few times but I’m still here because I’m just so passionate about it and I love it, but everyone processes and deals with trauma in their own way so I guess that would depend on them. So, yeah, finances. That’s probably the biggest thing to struggle with, and then dealing with the emotions that you have to deal with when you deal with these cases, with the animals and people that’s probably the hardest. Support, funding, and emotional trauma. Those three are probably the most difficult to deal with. I mean it all plays a part and it all can definitely be that, but when I think about it, I think how are we going to help these dogs if we don’t have the money, and then how are we going to help these dogs if we don’t have the fosters and support, and I don’t know just overtime having to deal with cases especially some that they don’t even make it, or they don’t even make it by the time you get them on the route once you put them in the car. That’s pretty devastating. Or not being able to help, like having so many cases being brought to you like us right now, where I have to say no certain people. That’s very difficult for me, and it breaks my heart whenever I think about whatever happened to that dog or cat, you know. So yeah, those are probably the biggest factors but number one for any rescue around the world is finances. Nothing happens without money. Nothing. You can have all of the love and compassion in the world, which is great. I mean you need that first and foremost, I get that. But from that standpoint its finances. I can’t fly a dog, I can’t bring it to the vet, I can’t pay for its food, I can’t without money.

**Follow-up:** Thank you for that answer. This again, might be a tough question, but what can be done to reduce or eliminate these challenges that you face in your work?

**Participant 3:** When we start spaying and neutering their animals around the world, one hundred percent. Government needs implement programs and policies to put lots of funding into them going around and trapping feral dogs and cats and spaying and neutering them and vaccinating them and rereleasing them. They do that a lot in different countries. Even Iran has that in certain parts where they’ll actually uh... I mean there’s all parts of the world, there’s people who are just not nice to animals and stuff but, in certain countries where you wouldn’t always imagine, they have that. They have these spay and neuter release programs that tag them with tags on their ears to say they’re safe. That they’re vaccinated, that they’re spayed or neutered. I think governments needs to induce policies all around the world for massive funding into their animal welfare programs and control is a huge thing. People just being more responsible and education. I think education and awareness is huge anywhere. And people just being more responsible. You could have all the bad breeders and puppy mills in the world which do not help, but if you don’t have people also educating themselves on not buying from these places if they happen to know even if they want a dog. Some just look past it. So, I think the biggest thing there too is just being responsible ownership, being thorough and knowing where you’re getting your animal from. I think for most, no matter how badly you want that animal from that particular person, group, breeder or whatever, and then spaying and neutering because you’re stopping reproduction, and obviously vaccinating because you’re stopping the spread of disease. So the number one things, spay and neuter, education and awareness, responsible ownership, and governments taking it seriously finally on how their own animals in their own country, becoming a more humane place for them. I also think that means inducing certain legislations and policies on animal welfare laws, animal rights laws, a strict prosecution, and strict sentencing for animal abusers. I think there’s a group of things there that those all would be huge factors for changing this world for animals, especially domesticated ones or the ones on the streets, cats and dogs mostly.

**Preamble:** From what you’ve described during this interview, it must be very challenging and demanding to work in a rescue organization.

**Question 5:** Can you please tell me what it is that keeps you continuing your work?

**Participant 3:** Passion, man. I just love helping animals. I don’t know, there’s times where you’re so down you want to quit, you can’t do it anymore and you just need a good night’s sleep or something or more. And I think even good rescuers quit and I could understand that so I don’t think that even when good rescuers quit, it’s not that they’ve lost passion; it’s just, each person can only take so much abuse, trauma, emotional distress, until they just have to walk away for their own personal health before they actually have a full mental breakdown and maybe never come back from it. So for me though, I’ve been through a lot of trauma in my life before I started helping animals so I was already good at coping with it or at least dealing with it. So, for me, it’s passion. If I didn’t truly love helping animals and feel like this was my calling in life regardless of getting paid or status. I don’t care about all of that. For me, to the core of my heart, I want to help animals for the rest of my life, and I truly know that and for me I feel lucky because I think I’ve found my true purpose in life. So, what is the purpose in life or purpose to life, for me it’s to have purpose. To truly love something so much that you’re able to do that throughout your life and make a difference. That’s an incredible thing. So for me, just me in particular, how am I able to continue, especially when I don’t even get paid, passion man. All day, pure heart. I just love helping animals. I get up because I need to do something with my life. I need to have purpose in my life. And that’s given me that. To the core of where I could literally pass away as a happy person knowing I have truly found my calling in life to what I wanted to do and never just set myself in a job just to be able to pay my bills every two weeks. And I went for it and I get to do something I truly love every day and I’m very grateful for that. But I also need to do something that’s very tough, it’s very hard. What makes me continue on, heart, passion, and I think support from my friends and family and other team members are huge. To do it as one, to always be there for each other so when you are going through those hard times, you have people telling you like “it’s going to be okay, were going to get through this”. So, I think that’s huge. Heart, passion, support. That’s definitely huge.

**Follow-ups:** What part of your job do you enjoy the most in your work?

**Participant 3:** That’s difficult. I always love being able to take on the animal when they’re in a really bad spot, and when you get them out you’re like “yes, we got them!” that’s such a rad feeling. And working with them is really rewarding but obviously adopting them out and getting pictures like weeks or months later of them just like, happiest can be with the family or at a beach or somewhere and you’re like this dog was tied to a chain for like three years left for die, barely fed, and this dog is now like…. you know? That beginning to the end story is just an incredible feeling, knowing that you somehow played a part in that dog’s journey for survival, for better life, you know, for everything. I think that feeling from when you get them out to when you see them at their final part, adopted and so loved by a family, how they should be, I think that’s an incredible feeling to have. That honestly makes it all worth it for me because I don’t get paid. So if I don’t get some sort of… I get payment, it’s just not monetary. I get a payment like that. I get a payment of gratitude, love, of just seeing the rehabilitation success. That for me is just like a serotonin bomb, you know? It’s just like... It just makes you feel awesome. For me, it’s just being able to do that.

**Follow-up:** In the perfect world, what would the lives of all dogs look like for you?

**Participant 3:** Dog’s that are ever only brought into the first home that ever into. That home being a very loving, responsible, caring home. You know, them being able to live long and healthy lives of love, benefitting their family and their people and themselves and enjoying life and all it has to offer. But I want to say that about any sentient being. I hope that every sentient being on this planet can be born into love, care, and compassion and live the longest life they can as healthy as they can. And when they do have to go, being surrounded by the ones that love them, so they never have to go feeling alone.

**Follow-up:** Those were all the planned questions, but from previously talking to you I found it interesting, about your philosophy about these “borders”, and I wanted to touch on that as well. Can you elaborate on your philosophy about “borders”?

**Participant 3:** Yea. Well, we all live on one planet. Planet earth. We’re here. Plain and simple. Borders are created by men. By governments, by men, by people, to separate different parts of this planet. And when they’ve done that, they can put in these ideals that some people are worth more or less than others, for the way they look, how they look, or their religious beliefs. To me, that’s absolutely nonsense. We are on one planet, we are one species, we’re all here together as one. And if we don’t work together as one to better help each other and to take care of this planet, we’re all going to go. It doesn’t matter if you’re Christian, white, black, or catholic, atheist, Islam, Buddhist, or whatever may have you, it doesn’t matter. Because generally to me borders are made up by men, and as soon as we create restrictions on them and put restrictions on helping each other around the world and benefitting each other’s’ humanity and the animals that live within that.

So, it’s kind of like to me that when someone is like “oh think of Canadian dogs first. Why would you import dogs when you have dogs here needing help?” You know what, I do get that. I get that from both spectrums. It’s just like how some people would say “why are you immigrating people when you have people here that are homeless needing help?” So, I get both aspects when they look at it like that, but I also think people have to look at it like… A border should never make anyone’s life worth more or less. We’re all here together. And especially for me, animals don’t really have a voice, right? They cannot explain to you really what’s going on to the general public in words unless you’re someone who understands animals just by seeing what’s happening with them and that’s how they speak to you. I guess the basic breakdown of what I’m trying to say is that, I think it’s ridiculous to think that any life is worth less or more because of a border. I think that when people try to tell me that “oh you shouldn’t bring in dogs from other countries because you’re killing Canada’s” that makes no sense. For one, some of the dogs we have here may not be the type of dog someone’s looking for. And that might be for someone else in the world. If you could save that life by bringing it here because it has no chance there for that life, for that person, that’s great. If I’m able to bring a dog in from anywhere around the world, from them going from a puppy mill to a backyard breeder, great as well. Because that’s a huge thing for us to decline that. Just the overall aspect of when people say that to me, I say “do you feel the same way about humans?”. So “if you don’t agree with dogs coming in here from other countries to be helped, do you feel the same way that someone shouldn’t come from their countries for a better life here?” and they go “well, of course not.” Well, why would that be any different? Why is it any different? We’re all living beings, we’re all looking for a better life, and some of these people were born into places unimaginable to us. I think some people haven’t travelled so they are a bit ignorant on saying things like that where if I took them to these countries and went, “you’re telling me you’re not going to want to help this animal on the street here right now? You have the opportunity and ability to help save this animal’s life, and bring it home to a great place, and you’re going to say no just because we’re in India? That dog means any less to you now?” so for me, that’s where I find it frustrating where people or like animal activists are compassionate, but they tend to put these limits on where they think animals should be helped or allowed to go and that doesn’t make any sense to me. I think with anything there, it is with moderation, of course if you’re just flooding 400 dogs from India to here every month and just willy-nilly putting them everywhere and they’re failing, ending up in shelters, getting hurt, yeah that’s never good. Everything has to be in moderation from how we allow people to immigrate people into our countries, how we immigrate to other countries, travel, same with animals. So, I think it’s both just both finding a really compassionate system in order to properly manage that within moderation. So, you want to import dogs, great, just do it ethically and morally, and make sure that you’re finding these homes or places where they’re not getting into trouble or they’re not leaving a bad perceptions on dogs coming from these types of countries, right? Like I get both aspects like if someone comes at me like “oh this and this” and I’m like “it’s true, there are people in Canada that are homeless and were letting people immigrate” but I think that’s just an aspect more as, one, then why don’t you direct more of your compassion for that towards helping people locally. You can do that instead of complaining on your computer that we’re doing that why don’t you get out and actually make a difference. Two, I think it’s up to governments to properly start putting funding for people who are suffering from mental health, or homelessness, the opioid epidemic. That is on the government. A lot is on the government because a lot of these people are suffering from a lack of funding for programs or certain things in different countries. Certain countries don’t even have welfare; we have welfare. Some countries don’t. Like you don’t have a job, or you’re disabled, good luck.

I think I’m trailing off now and kind of repeating myself. So generally, how do I look at that, I think every sentient being on this planet deserves a fair chance at a loving life and a compassionate life no matter where they’re from. I think every type of immigration nor import whether animal or human being or whatever can be done properly with proper ethics, moral or compassion and rules. I think it is important to always look at our own people in or own country to help them, but I think it’s always important to look at think about others around the world to open up your hearts to them as well. Because again, why am I different than you, why am I different than the person in India who’s suffering right now. Why is a dog on this street different from the one over there? To me they’re not. I just have closer access to this dog at this time but that doesn’t mean I don’t think we should care any less about other people or animals anywhere else in the world just because of where they’re form. That will never make sense to me but unfortunately that’s been the world since the dawn of time. But to me in my own beliefs it makes no sense. But I have also travelled 42 countries, just a fraction of this earth but I think when you’ve travelled and seen suffering and seen people and understood that struggle, and if you come back and have your own, I think it gives you a bigger perception on, we’re in this together. We need to be all be one and not just separate ourselves through borders, or beliefs, or the color of our skin, or how rich or poor we are. That’s just to me I’m never going to wrap my head around that. But that’s the world we live in.

I think there’s a lot of good out there but, yeah so for me importing dogs or cats, everything, as long as it’s done within moderation ethically and morally, and just more on the outlook of this is a suffering being. You have the opportunity to help it. That’s the question to me. If someone tells me that and I have the opportunity, why would I say no? I know I’m kind of going off on but this is a very sensitive subject for me because I have friends who only do local, I have friends that do both, I have friends that do only international, and you have everyone’s perspectives that comes around on this but I find most perspectives are only done from people that are not involved in that type of work. So that’s where I kind of feel that’s from. It’s almost like saying why are we letting people have kids when foster care is full. I think everyone just needs moderation, compassion, understanding, and then from a realistic point of view in the world we live in today, we have governments. And they control certain areas of this planet, so they need to be in charge of properly applying services to help but, they don’t. So it’s left on the people independently to try to make a difference. Which is just frustrating man. Did I answer your question or did I just go on?

**Follow-up:** Yes, this question was more of an improvised question from me so yes, your response was perfect.

**Participant 3:** Yeah, I’m passionate about that because I see everyone’s ways and I try to understand everyone’s beliefs on that but I also think there’s always room within all of that to help. So if you’re against import altogether, that’s a bit tough from me. but if you’re just against importing 400 dogs a month from India, yeah because I mean because who really can ethically and morally make that work properly and be there and support these people, so I think everything within moderation. Also, Canada we’re the second largest landmass we have so much room for people and other animals to come here it’s crazy. where others like the states could be congested, or in China where sometimes it’s harder to continue to build there. People have started branching out of those regions and the police are governments too, right? It’s not always the people as much as sometimes the government puts policies in where they allow people to kill dogs or to whatever, to overfish. You know what I mean right? So, yeah, that’s my random spread-across answer to that. You got me in a passionate subject. I just believe that we all deserve a chance and that we should have that chance regardless of these so-called lines made up by man because we are all on one planet. Yeah, we just need to start working together, this whole separation isn’t going to work for us to survive.

**Preamble:** I’ve asked you a lot of questions today, and I want to thank you once again for sharing your thoughts and experiences with me.

**Question 6:** Before I conclude this interview, I’m wondering if there’s anything I haven’t asked you about that you think it is important for me to know?

**Participant 3:** Umm, well regards to dogs or importing or rescue… Umm I guess whatever you’re passionate about, do it with everything you got. Do it with your whole heart, do it with everything and don’t let anyone tell you any different. Period. If you believe you’re doing just, far, and righteous, you’re always going to have people oppose that. Stand your ground, stay true to yourself and just never give up.

**Follow-up:** Right. Thank you. I will be ending the interview now

**P4 Interview Transcript**

**Introductory question:** Can you please remind me, from which geographic areas does your rescue organization import dogs?

**Participant 4:** Mexico, but in particular, from the central Mexico in a city called Guanajuato, in the state of Guanajuato, but I have taken a dog from neighbouring cities. Some as far as Mexico City and Guadalajara.

**Question 1:** Can you tell me about how your organization came to the decision to rescue dogs from these areas?

**Participant 4:** It happened sort of by accident I guess... If you want to call it an accident. I was travelling in the area and was really impacted by the mistreatment and the neglect of the animals there. It was a pretty daily sight we saw during our vacation, and I managed to connect with a person in the area that was doing some work in sterilization campaign and so from there it just kind of went step by step. I was volunteering, and then I decided to buy a place down there. And once I bought a house, the dogs just kind of showed up and from there I decided I had to find homes, and I didn’t feel comfortable placing them in homes in Mexico. So, I started finding homes for them in Canada and that’s how it started

**Follow-up:** So just to clarify, your rescue organization was originally focused on helping the dogs in Mexico when your rescue started.

**Participant 4:** Yes. When I first started I was raising money to fund helping the dogs in Mexico while they were in Mexico. That was through sterilization campaigns, but then it transitioned into rehoming, finding homes for them. Although I did continue to volunteer at local sterilization campaigns, but I just stopped doing that because it was too much to do, to do everything. So, I mostly focused on finding homes for dogs.

**Follow-up:** Thank you. Can you elaborate on how you got involved with... I’m assuming you have connections with shelters in Mexico, or rescue groups there. Can you elaborate on how you got involved with these groups?

**Participant 4:** First of all, I got involved right back when I started when I started with a local group called [Group name] and they focused mostly on spay and neuter campaigns and they tried to do some education so that’s how I initially got involved in the community. Then I gradually got to know local people, local Mexicans that were doing the same kind of work on rescuing, finding dogs on the street and I was also volunteering with the local municipal health department because they were having sterilization campaigns as well. So just through getting to know people in the local rescue community, that’s how I became more connected.

**Follow-up:** Great, thank you. I’m interested in this Push-pull dynamic in the rescue groups, where I’m trying to figure out if rescue groups are actively trying to get dogs from abroad or whether there are rescues or shelters in other communities that are trying to get the dogs out. So, when you started, from what I am getting is that you actively established these connections to get your rescue running, but as you are now more known, do you get more requests to help dogs in other countries or do you still actively look for connections?

**Participant 4:** No, I am not actively looking for connections. I have a lot of connections, I have more than enough now. But I do get requests from other people in parts of Mexico asking if I can, Mexicans that have found a dog or, other rescue organizations in Mexico either run by Mexicans or foreigners that would like to.. sort of.. connected or be partners to see if I can help and we can help each other, but primarily I have to turn everybody down because I have more than enough to do myself so usually passing on information to them on how they can move forward. I’m not sure if I answered your question.

**Follow-up:** Yes, you answered it perfectly. Sorry I phrased the question strangely.

**Question 2:** From talking to other rescue organizations, they often mention that they have factors they consider when selecting which dogs to bring over. Does your rescue organization also have that, and if you do, can you elaborate on that?

**Participant 4:** Yeah, for sure. Definitely I have a pretty strict protocol as far as what dogs can come up here. I do my best to evaluate them not only health wise, but behaviour wise. And, I have a health protocol. So all the dogs have their vaccines and they’re spayed and neutered, and I do blood tests and all that kind of stuff but I also try to get to know the dog as well as I can. Because of COVID it’s more difficult because I’m up here in Canada so I rely on my staff down there to let me know and I see them in action because they send me videos and stuff. But, I am actually going down there next week and I’m going to be there for a couple of months, and that’s what the way it normally is, is I’m down there and I actually live with the dogs. I see them on a daily basis, I get to see their behaviours, if they have any issues. So definitely any dog that has any aggression, it definitely will not make the cut. They stay down there, they either live their lives out there in the shelter or it depends on the severity of the aggression. It’s nice if they have a little bit of basic training but that’s not, definitely not something they have to have before they leave Mexico. So, behaviour and health. For sure, and more people are looking for smaller, medium sized dogs, so, yeah. Kind of, I’m not turning big dogs away, but they are harder to find homes for.

**Follow-up:** Thank you. You’ve already started touching on the answer for this next question I had prepared, but can you describe to me what a typical dog you rescue is?

**Participant 4:** Hah, typical? Oh my god. Is there a typical one? I don’t know if there is a typical. Do you mean as far as what they look like? Or behaviour, or personality, where they came from?

**Follow-up:** Uh, any kind of characteristics that these dogs might share, so small/medium sized that you already mentioned, or are they usually mixed-breed, or?

**Participant 4:** Uh, oh my gosh. I mean really, there’s all kinds of mixes. Mostly mixes. Once in a while, there’s a purebred dog and that kind of goes by what is the popular breed… you know the fad breed. Because they go through phases. Right now, it’s the Belgian Malinois; people are wanting them and huskies. So now you see a lot of those on the street. I don’t normally take the purebreds in because I know they will find a home in Mexico probably because there’s that.. people like them. But yeah, mostly from small to up to 45 to 50 pounds is pretty typical. They can be shorted-haired long haired. Most of them come from the street. Once in a while, I will take a dog from the animal control center there and… well, there’s no shortage.

**Follow-up:** Thank you. And can you describe what is life like for a dog before being rescued?

**Participant 4:** Hmm… What is it like before it’s rescued… Well, it’s a life probably running around looking for something to eat. And.. in the elements, probably being shooed away by somebody so that they’re not in the way. So, they’re not particularly wanted wherever they are. Sometimes a dog is in a situation where they are confined or tied up and then they are set free by somebody. Sometimes that will be the owner. Sometimes you will see a case where a dog is... I mean you don’t know how they would ever survive because they are so thin, but my theory is that somebody has had them like that and they just haven’t fed them and then one day they decide that it’s just… “we don’t want them anymore, so throw it on the street”. That’s when you see them when they’re absolutely… you don’t know how they survived. But yeah there’s a lot of risks on the road, on the streets for dogs. And there’s health dangers, there’s dangers of other dogs… being attacked by other dogs, and then there’s, you know there’s overpopulation. Especially the female dogs I really feel horrible for them. They’re the ones who have the puppies and you know, feed them. There’s also diseases that they can get form running free. There’s sexually transmitted diseases that they can get down there. You know skin diseases.

**Follow-up:** Thank you. That must’ve been hard for you to describe that so thank you. From talking to other rescue organizations, they also seem to have some procedure of finding the right match between dogs and the potential adopter. Does your rescue also have that, and if so, can you elaborate on that please?

**Participant 4:** Yes, we definitely try to make the best match possible. I have a pretty extensive application form which gives me a lot of ideas about what kind of dog they’re looking for, what their capabilities are, what they’re up for, in say training or house training. Hypoallergenic, they’re looking for a non-shedding dog, what kind of exercise, so yeah those are things I try to match the dog with the right people. And some people are looking for older dogs. You know, I get a lot of requests from older people, from retired people and also older people, 70, 80, even in their 90s looking for a dog. And they’re obviously not looking for a very active dog. So yeah it’s quite a process because you definitely don’t want to put any dog in any home because what’s going to happen is they’re going to come back to you. And that’s just more time consuming and problematic for everybody. And definitely not good for the dog because it’s going through yet another change. it’s not very helpful in their transition to a new home and their behaviour if they have any. If they have any milder behavioural issues. Fear issues, or reactivity is a big problem. Dog reactivity is a big problem. So yeah, you want to make sure the person who’s getting the dog is aware of the.. as much as possible about the dog. You want to know the person is up for it. Sometimes you have to be pretty blunt with people. You know, say “are you looking for a dog that’s house trained?”. A couple times I’ve had that happened saying “yes we want a housetrained dog”. I say sorry but, I can’t say they are house trained. So you do have to.. pretty good screening.

**Question 3:** What are the costs that are involved with rescuing dogs from abroad?

**Participant 4:** Hm.. Well, definitely depends on the dog. A big portion of the expenses are for transporting dogs, because of my particular area, we don’t have a lot of access to tourists that are flying back and forth. For example, dogs in Puerto Vallarta, you know that’s not about covid but, when it was normal life there were tourists flying back and forth all the time and they could escort dogs in their flights but because of where I’m located that’s just not an option. So normally I have to pay for either somebody to fly up and come back or, right now what we’re doing is land transport so, I would say I’m just going to look at my last transport thing because I always break down… So last transport I had 7 dogs come by land and my total cost for transport was $3,562, and the money I’ve put into the dogs as far as vaccines, spay and neuter, and blood-tests and all that stuff was $1,750. So my total costs out were around $5,300 for 7 dogs. And my adoption fee is pretty low, it’s $600 so, I actually took in $5,600. So I made technically I made $300? But that’s not always the case. Like the other one is total value expenses... yeah expenses were $3,400, and adoption fees were $3,600… Is that right… Oh, but this one was a loss of $1,242… So yeah, there’s always a loss. The rest of it is covered by me in my own money that goes into it.

**Follow-up:** Right, and apart from your own money, how else do you recuperate these costs that you are putting for rescuing.

**Participant 4:** Well, there’s the adoption fee and I do have monthly doners. And sometimes people will just donate say, $50, $100. I did have a GoFundMe page last year and that did really well. A few.. Three years ago I think I had a fundraising event so it was a solid option with some music and people bought tickets. So, yeah but that’s just another thing I have to do and I just don’t get around to it.

**Follow-up:** Has there ever been a time where you couldn’t rescue a dog from abroad because it was too expensive?

**Participant 4:** No. Too expensive?

**Follow-up:** Yes

**Participant 4:** I don’t even think about that when I rescue a dog. I just take it. I don’t think about that.. I just take it and I’ll cover it if I have to.. Like you know, I couldn’t turn it down if I knew it was going to.. In fact, that might be a reason I would take the dog, is because somebody is trying to rescue a dog and they can’t, they don’t have the money themselves. You know, that’s a big problem in Mexico they just don’t have the money for certain treatments. So, I would take a dog for example that has TVT, I don’t know if you’ve heard of this through other rescues… It’s a transmissible venereal tumor and dogs get it if they’re stray dogs. It’s kind of a chemotherapy but they do get over it. It’s a weird kind of cancer but it doesn’t come back. So, there is costs for local rescuers.. It’s very difficult for them to cover those things so I might take a dog in like that or if it’s some, you know, dog that’s in really horrible shape.

**Question 4:** Tell me about some challenges you face through your work?

**Participant 4:** Hm.. challenges would be… Well, there’s basically you know the finances. That’s always a challenge. But like I say, I will if I have to, I will cover it up myself. There’s the transport, is a challenge because it’s everchanging and because of the cost of the transport and finding people to travel to escort the dog on their flights. Another big challenge is that it’s a never-ending job and that can get to you. There’s a lot of mental stress and physical stress, there’s not enough volunteers. Let’s see I’ve just made a few notes here... And once in a while there’s a person who doesn’t really understand the challenges of a dog, and what’s required of them when they do get a dog that is rescued. As far as their, the time they need to transition and, I mean it’s not a serious challenge. The most is the finances, and the mental and physical stress, emotional.

**Follow-up:** Is there anything that could be done to reduce or possibly even eliminate these challenges that you face?

**Participant 4:** Probably, for me in particular, it would be is if I had more people, more volunteers to help. Yeah, that’s basically it. And more money.

**Preamble:** From what you’ve described during this interview, it must be very challenging and demanding to work in a rescue organization.

**Question 5:** Can you please tell me what it is that keeps you continuing your work?

**Participant 4:** It, well it’s the happiness at the end of the tunnel. When you see a dog that is, transformed from.. You just see so much potential in the animals and you see how happy they can be and just.. It’s just amazing to see them when they’re in a good home when you have seen them.. you know, just as a discarded creature. And also seeing the people that are so happy to have a dog. So, it’s that. Those two things. That’s it.

**Follow-up:** Thank you. And what do you enjoy the most in your work?

**Participant 4:** Hmm. It’s the final result is what I really enjoy. I really enjoy seeing an animal in its progression from say it’s a really sick animal has a really bad skin condition. I just really love seeing the transition into a healthy animal. I mean there’s all parts of it I love, but sometimes there are things that are very challenging day in and day out. So, you know it’s very overwhelming at times. I just love seeing the animal go to great homes.

**Follow-up:** In the perfect world, what would the lives of all dogs look like for you?

**Participant 4:** It would be having a family, having a good home, given the love and training they require, guidance. Not so much training but guidance I guess. Having a shelter and having good food. And having no dangers.

**Preamble:** I’ve asked you a lot of questions today, and I want to thank you once again for sharing your thoughts and experiences with me.

**Question 6:** Before I conclude this interview, I’m wondering if there’s anything I haven’t asked you about that you think it is important for me to know?

**Participant 4:** Hm.. Umm, well I guess. I feel that it’s important that people continue to help dogs that are in need. I don’t want to go into uh... My personal opinion is that I just feel that there’s so many dogs that need to be, that are already existing that need homes. And I wish people would think about that before going and buying a dog.

**Follow-up:** Great, thank you for sharing your thought. I will be ending the interview with that closing thought.

**P5 Interview Transcript**

**Introductory question:** Can you please remind me, from which geographic areas does your rescue organization import dogs?

**Participant 5:** From Alabama.

**Question 1:** Can you tell me about how your organization came to the decision to rescue dogs from there?

**Participant 5:** Yeah, it’s a very long and convoluted story, but we looked for regions that had high rates of euthanasia, were near a major international airport, and where we had trustworthy colleagues on the ground to assist us with transportation and assessment. Trusted shelter partners.

**Follow-up:** Right, so just to clarify, does that mean that your rescue organization, from when you started your rescue, was focused on rescuing dogs from Alabama?

**Participant 5:** No, we started out rescuing from California, same criteria, but I had a friend there who was a dog trainer, so we enlisted her in assessing dogs that would be suitable for our program.

**Follow-up:** Can you elaborate on how you got involved with these trusted connections that you have?

**Participant 5:** From California?

**Follow-up:** Sure, and also Alabama. Just how you got involved with them.

**Participant 5:** The longer story is that I had been looking for a dog for a long time, and I ended up being referred to an organization through the internet in Louisiana. And it occurred to me then, I did some research and realized that there were huge amounts of dogs dying in US because of the lack of homes. So, I did a little bit of research myself and I could probably logistically do a half decent job in saving some and bringing animals in. I had a colleague who was a flyball champion, and one of her friends was a dog trainer in Fresno California. So, she hooked us up and we decided to put together a bit of a program so that she could pull from, they have a shelter in Fresno that was high kill at the time and so we agreed to work together to try and save some animals.

**Follow-up:** Okay, and just to clarify, was it you who reached out to them?

**Participant 5:** Yeah, I can’t remember exactly how it took place but I think my colleague, wrote to the lady and me, “Hey I’m hooking you guys up, [Name] is interested in starting this rescue, would you be willing to help”.

**Follow-up:** Right. In this interview as well, I’m interested in the push/pull dynamic between the rescue and shelter in source communities.

**Participant 5:** So, the problem that we had there was the shelter was not rescue friendly. At the time the man told my colleague directly he would rather euthanize dogs than send them to rescue. Umm, it was cheaper and less hassle to kill the dogs than to find spots for them. So, we did have a real issue in that. The fact that she was a dog trainer of course did help. She had some professional associations. What happened then was, though as soon as of course people figured out we were rescuing in the area, we had tons of people come to her. Other trainers, like “oh my god I found this dog can you help me out?” right? And so, it’s sort of that thing once you sort of have forage in an area, that has a really high kill rate and a real serious problem finding homes, you get inundated and then you have to make some tough choices.

**Question 2:** From talking to other rescues, they often have factors they consider when selecting dogs to bring over. Does your rescue also have that, and can you elaborate on that?

**Participant 5:** Yes, so we set up clear criteria. I should just tell you that because our board contains lawyers and most of us are professional women, we had some pretty strict criteria setting up. We still have the same board pretty much as we had for years. So, were a small organization but I would say that out of the most organizations we probably have the most rigor around our criteria and our financial processes and such. So, we were very clear that we would not rescue dogs that were over-represented in the B.C. shelter system. So that includes pit-bulls, rottweilers, other large breed, huskies, those kinds of things. So, we made an agreement that we would not be rescuing those dogs. That our primary focus was on small to medium animals, but we would take larger animal’s temperament-wise. But we would not rescue animals that were unsuitable for family placement. So, we don’t rescue three-eyed one-legged dogs that bite for example. So, they had to have suitable temperament, they had to be healthy. And they, had to be, past some basic testing in terms of… Like I could deal with some behavioural modification, but if their behavioural issues were such so that they had major separation anxiety, leash reactivity, resource guarding, we would refuse to take those kinds of dogs. And the big thing is that they had to be social with other animals.

**Follow-up:** And those are for cats?

**Interviews:** Dogs. They had to be dog friendly. Lots of people don’t have cats, but they had to be… Because particularly Victoria is a dog friendly city, dogs go everywhere here. We hike off-leash with our dogs, the expectation is that everybody’s dogs can actually get along with other dogs.

**Follow-up:** Thank you for elaborating on that, I was not aware. From what you were stating earlier, I think I’ve already started to get a sense of the answer to this next question, but can you describe to me what a “typical” dog that you rescue is?

**Participant 5:** Yeah, we tend to take the dogs that are readily not adoptable in the local area, so let’s say you’ve got some nice poodle that comes into the shelter. Somebody’s going to take that dog, right? So, we try to take dogs that were overlooked for adoption in the shelter as well. So, dogs that were black, for example. Or, some dogs that just maybe had some medical issues, they needed dental work, that kind of thing. We look for dogs... my personal… its funny we all have our favorite, but I tend to like dogs that are medium sized, that are reasonably athletic, good hiking partners, stuff that would really fit in with our Victoria type people. I also rescue a lot of seniors because there’s a lot of seniors here in Victoria and they look for senior dogs. Again, they tend not to be adopted very quickly in the shelters. They almost always require extensive dental work or other medical intervention. So, we tend to take those. I tend to take... I like a lot of the terrier types. Like small little Jack Russel-y things you know, little pig mixes, you know, just general mixed-breed dogs. We do take purebreds, but again, we tend not to take many of those because there’s lots of purebred rescues that will take them. So, we like to be sort of the, not the rescue of last resort, but it’s all about personality for us. So, you know if the dog’s got a... and I’ve taken dogs who are ugly as sin but had the most winning personality, that’s my favorite. I love to take those kinds. So, generally they would be mixed, you know, I call them “apartment-sized labs” so, smaller lab-mixes, terrier-mixes, some chihuahua, because that’s overrepresented down there as well. And schnauzer-type dogs are hugely popular here, so. Again, I emphasize it’s more bout personality than breed.

**Follow-up:** Thank you. Can you describe to me what life is like for a dog before being rescued?

**Participant 5:** Yeah, some of the dog that we take have been in really bad shape. Um, we’ve had dogs that have been locked up in houses and abandoned and been near death. A lot of our dogs are brought in by animal control to the shelter so they’ve been dumped in remote areas, they’re usually parasite infested, lots of flees of course, demodex, starvation. I would say that majority of our dogs are strays. We do take the odd dog in where it has been well loved and the owner has passed away and the family doesn’t want it anymore. I love taking those dogs in because they’ve already lived in a home, and they’re usually well trained and well-cared for. So yeah, a lot of strays. I mean some of them are in pitiful shape. We tend to take probably more medical cases than some other rescues, so I took a dog that’s been hit by a car for example. Because you know, it has an undisplaced hip fracture, it needed crate rest for weeks. I can make that happen, otherwise it would’ve been euthanized, nobody else is going to take that dog. So yeah, we take in dogs, like I said, who have been very poorly cared for. The area is exceptionally poor, so.

**Follow-up:** thank you. From talking to other rescues, they often have aa procedure of finding the ‘right match’ between the dog and the owner. Does your rescue also have that, and can you please elaborate on that?

**Participant 5:** Yes. So, we do have an application process. So, and there’s probably two parts to it. But, so, we have an application process. You fill in the application, we ask a whole plethora of questions, I could certainly send you the application if you are interested in it. But really what I am looking for in that process is looking for how much time people are at home, how much experience they have with the dog, and how active they are. Right, so I’m not going to place a Weimaraner mix in a home where they only walk 20 minutes a day, right. So, it has to… I would say it’s like computer dating. The basics have to match before you can even move to the next step. We then conduct an interview with the individual, see what their homes like, I would ask certain questions depending on the dog that I might have in mind for them. If they got a fenced yard, all of those criteria that we look for. And then we do a foster to adopt program. So, they usually take the dog for two weeks or more depending on how long they need, we provide all of the things the. Dog needs for that period, so, vetting, food, medication, anything else they need. And we also provide training if they need it, right. So, I do have a trainer, on staff, well not staff but on call, who basically if they have any even just small little problems that needs some advice, we can loop them in. And then once that process is gone and they decide that the dog is theirs, and they want to keep it, then we go sign on the dotted line and basically finalize the adoption. Now, I do things slightly different to, and I guess it’s bragging a little bit. We have a really good reputation. So, a lot of people come to me and say that they want me to find a dog for them. So, I have a guy who basically trains dogs for veterans for example, for PTSD. I look for dogs for him sometimes. Again, I’m looking for temperament. Or I have an old lady who wants a Yorkshire terrier mix, I will look stuff for her. A lot of our dogs are not advertised, I interview the people in anticipation of finding them a dog sometimes. And then I will actively go and look for an animal that meets what I think might meet their criteria.

**Follow-up:** That’s such an interesting procedure and you’re doing things differently from other rescues, so that’s really interesting to hear.

**Participant 5:** Yes. The best thing about that is, that I can usually, if the dog’s been assessed well and I’ve assessed the home well, I can put that dog straight into that home on a foster basis, right? There’s no obligation, takes this thing out of it for people and the dog doesn’t have to get moved 14 times, right? So, it gives the dog an opportunity to just get its legs under it, and we provide support ongoing. Sometimes I go walk with them, right? So, I’ll say “hey listen let’s go up for a walk on Wednesday, I want to see how he’s doing with you” that kind of stuff. And that’s the benefit of being a small rescue, right? And so, yeah, I would say that at least half of our adoption are probably done the other way around, the people find me first and I find them a dog.

**Question 3:** What are the costs that are involved with rescuing dogs from abroad?

**Participant 5:** Yeah so, the most significant cost of course is vetting and transportation. So, most of the rescues have vetting packages. Which are fairly, you know, you pay 100 bucks for the dog, and it gets neutered and gets vaccinations. We don’t like that, right? We pay over the odds for our vetting because there’s certain processes that I want. So, for example, every one of my dogs get a fecal screening, right? I say, “do a full parasite test”. Even if it comes back negative, they still get full worming processes. They also do heartworm testing, right? We also, I will vaccinate them for Leptospirosis which isn’t part of their normal plan. And sometimes there’s other things I want done to the dog. So, for example I just rescued a dog this week, large, flat-coated retriever. He’s got a big gouge on his leg, now they would just leave that, right? But no, I want the vet to go in, I want him to, you know, clean that up, close the wound properly so that the dog doesn’t have any bald spots or scarring, or suffering right? We also pay for analgesics when our dogs get spayed. They often don’t, right? They don’t give their dog any analgesics. So, there’s a few things that we pay for extra. I’ll pay for dental costs, ear cleaning, grooming, all of those kinds of things if necessary. So, there are extra costs for that. The biggest cost for us, of course, flying... We fly our dogs, so I won’t have them... Some people transport them over 5 days or something ridiculous. I know a lot of people do that and I get why. We fly our dogs so there is a cost. Although, having said that I do have negotiated rates with [Airlines]. All of our dogs’ fly [Airlines]. And so, I do have special rates with them. And then the other cost, Kai, is really kind of, interesting, it’s equipment like crates. So, we have flea medications, and crates, so, so a crate to ship that large dog is going to cost me $150 US Now, I do sell them on, but nonetheless right, its used by that point. We put them on heartworm prophylactic, right? So, all of the dog gets a broad spectrum murmur, they all get defleaed, they all get prophylactic heartworm meds. And the actual cost of transportation isn’t just flight, right? There are import taxes, there’s [Company name] are the broker for [Airlines company]. It’s $100 per shipment just to simply have them sign the paperwork. Right, and of course I have to get them on the ferry, so I drive over there and pick them up. So, even though the dog may technically have only costed $100 from the shelter, by the time it gets to me, probably you’re looking into the $500 range.

**Follow-up:** Right. And how do you recuperate these costs.

**Participant 5:** Adoption fees, and fundraising.

**Follow-up:** Okay, thank you.

**Interview:** So, we charge $499 for adoption, we’ve had to increase our costs a little bit. And then we do other fundraisers. So, we sell calendars every year, we do a scavenger hunt, we do online auctions. A host of things.

**Follow-up:** Has there ever been a time where you couldn’t rescue a dog from abroad because it was too expensive? Why was that?

**Participant 5:** No. But we are probably, fiscally we are very conservative. Right? We also donate to other rescues sometimes. So, for example the shelter that I rescue from, they often run out of food for animals. The city only has a limited budget and so all the animals will be euthanized if they don’t have food. So, we provide food for them. We provide vaccines for them. We fundraise all of that money.

**Question 4:** Tell me about some of the major challenges you face through your work?

**Participant 5:** Umm… So, in terms of… The transportation has been problematic particularly with covid. I’ve had to cross the border which is not my idea of a good time. Transportation Is probably the biggest challenge because there’s no direct flight from [State in the United States] to Vancouver. Right? So, the dogs have to overnight in Seattle, I’ve had to find kenneling, people to pick them up and drop them off. So, if were to put it to words, the logistics of getting them from there to here is very, very challenging. I would say that’s the biggest challenge we have. Finding people on the ground there, on the ground here, to be able to facilitate all of this and having all of the airline’s schedule to match it, is pretty tough. That’s the primary challenge.

**Follow-up:** Right. Are there other challenges you would like to share?

**Participant 5:** Timing. We’re fortunate that we have great quality homes here, but I have more people wanting dogs than I have dogs.

**Follow-up:** Is there anything that could be done to reduce or possibly even eliminate these challenges that you face?

**Participant 5:** I think, there needs to be changes to the regulatory framework for animal importation. And I’m not talking less restrictions, I’m talking different restrictions and even more restrictions in some ways. So, I think we need to make it, not less difficult, but for example you can’t import puppies. Legally, anyways. Lots of people do, but you’re not supposed to import dog under 8 months of age unless you have an import permit. So, the rationale is to protect the public, but how does it protect the public. If we were to import more puppies, A) we would be able to two per crate, because you can put two puppies in a crate and ship them; so, it saves a lot of costs. Plus, it potentially would save more lives we could get more dogs through for the same cost. So, I think changes to the regulatory framework could facilitate better control and still allow for more importation. Some of the things are just going to be out of control, I can’t change [Airline]’s schedule. I mean I’d love to coordinate a direct flight between [State in the United States] to Vancouver. I’d be bringing in dogs every week. But that’s just not going to happen. Umm, I don’t know. I guess better cargo… Increased cargo capacity I suppose, could be one thing.

**Preamble:** From what you’ve described during this interview, it must be very challenging and demanding to work in a rescue organization.

**Question 5:** Can you please tell me what it is that keeps you continuing your work?

**Participant 5:** Um... I think the fact that there’s such a desire for animals here and you see how meaningful they are for people and how few there are here for adoption, right? So, people are desperate to get dogs and they’re buying dogs from disreputable breeders and other places where animals are being mistreated. I mean if you can’t meet the demand, people are going to go, and buy from backyard breeders and that’s really what I don’t want. There’s something morally reprehensible about having... euthanizing millions of animals and breeding the same amount at the same time. Like it just doesn’t compute right? We need to do better. And I think that part… It is my commitment to the moral and ethical treatment to the animals that I personally hold value. Um, the other thing I think is seeing... I actually just went for a walk last night and saw two of my dogs. Seeing how much joy, and love they bring to their owners is certainly self-reinforcing. Right? It’s lovely. And of course, you know, seeing the dogs live long happy lives. I mean now, unfortunately a lot of our dogs are dying, right, we’ve been in business for 15 years. A lot of them are passing away, and we’re seeing repeat clients, right? A lot of people coming back and saying, “my dog passed away can you find me another dog?”. I mean certainly that. Seeing the conditions that they live in, and I think the… I think if you really dive into it, as I do, right? I go down there, right? I go there. I volunteer at the shelters, I put animals to sleep. Umm... I have been asked to help them with some of their organization… I came up with the algorithm for euthanasia for example for one of the shelters to help them make appropriate decisions for the care of their animals. When you see the conditions in which the animals live, and the toll that it takes on the people who work there, I think you’re compelled to do stuff. To do something.

**Follow-up:** Thank you. And what do you enjoy the most in your work?

**Participant 5:** In some ways I actually enjoy the rescue part of it. Like the organizational… Like getting it all in line... Like fitting the pieces to get them here. So, you know once you land like 12 dogs here, it’s like “Woo! We did it”. I think for me, I don’t really like the personal acolyte stuff, that’s not really what drives me. I don’t ever want to be on TV or on a website or the news, God forbid. I go to the park and see some of my dogs just enjoying life, and that’s rewarding enough for me. I also think that the joy they bring to people, sometimes we fail to realize that… for example I tell a story about an older fellow who had a stroke, his wife adopted a lovely little dog from me. She phoned me up one day and said how much joy the dog had brought to her personally because he was in an advanced stage of dementia and the dog had brought such joy to her husband and she said “you know, I have to tell you, the dog was sitting on his lap and he was talking to me the other day”, and I said “that’s lovely!” and you know, she said “but you know he hasn’t said anything for two years”. Right? And so, dogs as a therapeutic thing for humans is also something I find very rewarding.

**Follow-up:** Thank you for elaborating on that, that’s really heart-warming. In the perfect world, what would the lives of all dogs look like for you?

**Participant 5:** Well, they would be well exercised, well-loved, and well cared for, obviously. And have safe spaces to run and be dogs. For me, even the smallest chihuahua should go on a two hour hike every day, right? You know, so, um, yeah, I think that, again, they would be provided with appropriate vet care, food, exercise, yep.

**Preamble:** I’ve asked you a lot of questions today, and I want to thank you once again for sharing your thoughts and experiences with me.

**Question 6:** Before I conclude this interview, I’m wondering if there’s anything I haven’t asked you about that you think it is important for me to know?

**Participant 5:** No, the only thing that I think again that is something... That I’m struggling with a little bit is to figure out... I mean there are lots of rescues out there who are not great. They’re well-meaning and lovely people that they are bringing animals but are dangerous and have diseases. And we need to stop that because I am worried about the impact of that on... It’s a reputational risk for all rescues even those who perhaps go above and beyond. So, for me, the biggest and most important thing, and of course as an importer everybody thinks you don’t want regulation, I want more regulation. I really do. I want all dogs to be tested for parasites before they come to Canada. I want all dogs tested for heartworm. All dogs should be going through a temperament test process. I think it’s easy for us to suggest the importation of dogs is inappropriate when people aren’t doing their job properly.

**Follow-up:** Right. Thank you for sharing. With that, I will be ending the interview by reading a script I have prepared.

**P6 Interview Transcript**

**Introductory question:** Can you please remind me, from which geographic areas does your rescue organization import dogs?

**Participant 6:**

We rescue both locally in Canada as well as importing. So, we mainly have rescued from Mexico, Puebla Mexico to be more specific. And, Los Angeles (LA) area. Those are, to my knowledge only two non-Canadian areas we have taken in from thus far.

**Question 1:** Can you tell me about how your organization came to the decision to rescue dogs from [geographical region]?

**Participant 6:** Yeah, so I am the founder of [Rescue organization] and I became aware of it about 13, 14 years ago when I was a foster home for another organization in Victoria. And she had imported dogs from the LA area. So, I became aware of just the need of their shelters versus ours. Okay. Walking through, one of our main motivations of importation is walking through one of these high kill areas versus our shelters. So that was kind of my, one of my eye’s opened to that. The first rescues I did through “[Rescue organization]” I was actually at the time in investment banking, and I went down for a mutual fund conference. And my boss and I were both very passionate about dog rescue. And on our trip we brought back two dogs, and put them for adoption and then thought “let’s keep going”. So, that’s how it began and.. sorry I don’t know if I answered the big question.

**Follow-up:** That’s perfect. So, just to clarify, does that mean that your rescue organization was focused on foreign adoptions, and rescuing dogs from foreign countries?

**Participant 6:** Yes.

**Follow-up:** So I’m assuming you have shelters that you work with in these source communities.

**Participant 6:** Yeah, so for most American shelters you actually need to work with another organization not the shelter specifically. I’ve only found one shelter that would work with Canadian entities specifically. That is because quote on quote what’s called “Pull”. They want you to have basically the same as being a registered charity here, but they want it from the US So basically, someone to be accountable for whoever is taking these dogs. So you can’t as a Canadian entity directly pull from the shelters. So, you work with another organization.

**Follow-up:** Wow, I had no idea about this. So, you as a Canadian rescue, works with a rescue organization in the US that works with shelters.

**Participant 6:** Yes, correct. That is kind of the easiest way to operate and make sure things are done on the ground there and so on. And our Mexican rescue, is quote on quote the shelter/rescue directly.

**Follow-up:** Right. Okay, that makes sense. And can you elaborate on how you got involved with these rescues or shelters when you began working in the rescuing field?

**Participant 6:** Yeah, so originally, I was first rescuing from LA. Again, because I saw the need there being from [Area]. And honestly when I started out, not knowing a whole lot about the need in Canada, or even knowing there was a need. And now that like I said we… So Canadian areas we have rescued form mainly is Manitoba. Because we have built connections there with some very dire needs as well. So, there is Canadian need. It’s just not in our face and as apparent as some of these other areas, right? So, in some ways I would’ve loved to work with local Canadian shelters but one, didn’t want to work with a small guy they’ve never heard of. So, my drive to help dogs in need was there so I started networking… I can’t remember how I originally found this woman named [Name] but I started working with a passionate volunteer in a shelter that I was going to be working closely with, and they had heard that She was familiar with bringing dogs to Canada, so transportation and all of that. So, they connected with me and her. So basically how I found these connections is just networking with quote on quote, the dog rescue world, which is operated on Facebook, basically. At that time. I think now Instagram is definitely networking tool as well, but I don’t think Instagram was a thing when I started. I still think, in my opinion it’s Facebook. I’m old haha.

**Follow-up:** So when you started your organization, you were actively networking,

**Participant 6:** Yes.

**Follow-up:** but now that you are more of an established organization, do you still network for connections?

**Participant 6:** Yes, and no. So we, from the grand scheme of it and probably who you are going to interview, we are very small. Our… if you look at our numbers, we aren’t a numbers guy. We’re not doing hundreds of dogs a year. We’re not doing the volume of which other rescue organizations do. That is because we are small and we seem to work… every time to roll, the world is actually we had a location in Calgary, I was living there as well. Moved back, we were going to continue it, it kind of crumbled. Every time we’ve tried to get too big, we’re kind of control freaks, so every time we’ve tried to get too big, um, we don’t like how it’s operating. So, we stay very small. Another kind of way we’re different, I was in investment banking quit my job when we moved to [Canadian Province]. became a dog trainer. Force-free dog trainer. So, really had an education on how I can help these dogs more, than I was. And learning a lot about the force free movement. But anyways I digress. Do we network continuously? Yes. Because there’s a high rate of burnout in rescue. So [Name] I told you about who was my first contact, she kind of took a break from a while from rescue. And quit for a little bit. I know she’s back at it but she’s doing a completely different realm in rescue. So, yes we’re continuously networking. People move on. One of my other great contacts, actually just used… She was from Germany, she married an American, with COVID and stuff she went back to Germany. So, she left. So there’s certain connections we try to rebuild. But we like to stay small. We actually get contacted more from overseas help. In our email alone right now, there’s three requests from all over the place. And we haven’t gotten back to them just because were trying to find our groove of what were comfortable doing.

**Question 2:** Can you tell me about the factors you consider when selecting dogs to bring over from these areas?

**Participant 6:** Yes, the main one for us again, so, we have made it a priority to educate ourselves with dog behaviour. So we consider ourselves… There’s a lot of rescue that are well meaning, not well educated. That’s like the simplest way I can say it. So, because of that, we will take in certain behaviours but because we are small and run by dog lovers and rely on foster homes which are also all dog lovers, 90% of our foster homes have dogs in them. I would actually say higher than that, so dog friendly is number one. So we like to see, especially with large dogs, we want to see dog-to-dog video interactions. We want to see... read their body language. It’s very important for us. Some slip by. Can show well in a shelter environment, and that has happened to us a few times. Because they’re basically flooded. I don’t know your program but they’re basically you know “just going to behave, I don’t want no trouble” but when its more one on one, then you start seeing behaviours come out. Because of that and amount of intensity it takes, we basically make a rule, we aim for dog friendly. So that is the number one rule. We do ask about bite history, whether it be two dogs or one people or what not. The level of bite. And we assess video when we can, especially with large dogs. We try to make it a rule, but the problem is some shelters, especially the American shelters, you’re dealing with volunteers only. The staff don’t go over and beyond… They have their own job to do, and working with Canadian rescues aren’t it. So yes, our criteria, to simplify the question would be, dog friendly, as much as we can tell is number one. And then we kind of assess and were very conservative in what we can help whether medically or behaviourally.

**Follow-up:** Right. And in terms of breed, would that not really matter?

**Participant 6:** I try not to know no limits with breed, although I would say we also mandated, we’re not a high-energy rescue. So, we kind of have… there’s one breed we’ve kind of written out in regards of we’ve had experience, they’re not for us, is huskies. And that, is just because we are not... the structure of the kind of foster homes we attract and what we do, we’re actually geared towards more like seniors, medical cases, pits have a high need everywhere, so, we kind of go to… or attracted to more in need, and not too high energy breeds.

**Follow-up:** Great. I accidentally may have already touched on the answer for the next question I had planned, but I will ask anyways. Can you describe to me what a “typical” dog that you rescue is?

**Participant 6:** That… So, there is no “typical” dog. But like we’ve said, we definitely are attracted to... The answer to is… Always in the back of your mind in rescue is “what’s adoptable?” but sometimes we don’t care. Like for instance I went on a rescue trip yesterday, picked up two dogs that came from Mexico. On paper these two aren’t adoptable… On paper, these two aren’t adoptable. I feel like one is, she is amazing through and through but has a broken leg, and it healed that way. So, we’re coming out of the choice now, do we amputate it or leave it as is. And that’s going to be assessed by our vet here. The vet in Mexico said, “leave it, then make our decision here”. The general dog, there is no... we are an open door, closed dog aggressive, closed overly high-energy.

**Follow-up:** Thank you. Can you describe to me what life is like for a dog before it is rescued?

**Participant 6:** Various backgrounds as you can imagine just like with people. Uhh… The majority that we rescue from, and again, LA and Mexico kind of has two different answers to it, but not really. So, basically living on streets. Subjected to Mange, finding food, finding shelter, finding water. Can be from abusive situations, can simply... a lot of those areas that we rescue from are financially… people have issues themselves so it could be a medical issue that people couldn’t pay for and then relinquished it to a shelter. A lot of dogs from Mexico, two we just picked up were both found as strays, one could barely move under a car, tumors, broken legs, geriatric, severe dentals, holes on their mouth, various.

**Follow-up:** It seems like they all had some sort of challenge in the past.

**Participant 6:** Yes. And were talking specifically to… and again Manitoba has a lot of what we… One of the rescues we work with there, she said… I couldn’t understand a situation I said “why won’t these people load this dogs up on this plane?” she said “it’s like dealing with a third world country. We are living in a third world country, essentially, when it comes to the rescue mentality”.

**Follow-up:** Interesting. Can you describe to me what life is like for a dog before being rescued in Manitoba?

**Participant 6:** I can give you a great Facebook page to check out. [Rescue Name]. And they do a great job of… Actually, it was really interesting when they had the fires there, they were going to help bears, giving them water, the footage they had was amazing. Yeah, they’ve got some nasty stories so check them out.

**Follow-up:** Okay, wonderful thank you for sharing that. From talking to other rescues that you had some procedures to find the ‘right match’ between the dog and the owner. Does your rescue also have a protocol, and can you describe to me?

**Participant 6:** Yes. Our protocol, we’re always dog focused. Especially me being the founder, I’m kind of the meanest of them all. We don’t keep in mind the people in general at first. We rescue the dog, we assess the dog, we are very clear in our write-up the dog’s needs, etcetera. From then, people apply, then we look at those applications. So, we have actually no longer, we even say, if you are not on our application, if you are not applying for a specific dog, please reach out to us if you do see a dog that interests you. We don’t match-make any longer, apps that come into dogs. It doesn’t work for us. It seems like they don’t read, they’re just attracted to the picture, and "that still happens but I’ll reach out and say, “hey this applicant (prospective owner) would be really awesome for this dog”. It turns out that if that wasn’t the person’s choice, it rarely works. And we just found that, so our process is. Here’s the dog, here’s the dog’s needs. Applications come in, and we look at who are the top one, two, three (prospective owners) that fit’s the dog’s needs. And then we will do a phone, Zoom type interview, and then start the process there. But it’s always dog focused, we no longer try to quote on quote matchmake.

**Question 3:** What are the costs that are involved with rescuing dogs from abroad?

**Participant 6:** Okay, so… Hm…. We’re eight years old with a high sense of wanting to learn and educate ourselves and do things right. I can say, when rescue start out, and when we started out, there’s a great desire of wanting to help but not necessarily with doing education behind you. So, that’s where you see trying to turn dogs and… We’re rescuing from sometimes desperate situations. And depending on the desperation that the people living on what I call “On Zero”, sometimes they will cover all the costs. Right? When that starts happening though, people here start taking advantage unknowingly. So sometimes the rescue will pay for a lot of the way, whoever you’re working with. If you look around closely you can find that. But that’s not fair also. So depending on who we work with, I know our Mexican rescue, they take in dogs, we kind of work with them to then see what is the right fit dogs for us, and they cover most of the way and then we pay them a quote on quote “pull fee” plus their flight from Mexico. So that’s our arrangement we have with them. So the pull fee is… I have a girl who’s coming back from whistler today, she does all of that, and I think we probably pay... give the shelter 300 to 400 a dog, probably around $300, and we also pay for their flight. We’re coming to this weird…. Our fees to adopt are no longer covering all our expenses. So we kind of have to look at upping them. But we also look at where rescues in the area, what they’re doing. So its hard right now to manage the books so we also greatly rely on donations.

Umm, it depends. Transportation costs, medical costs, it depends who you’re working with, and what the expectations are. We cover tons of medical before they even arrive into our care, and again once in our care, we also… and this might be a question down the road from you, but a lot of rescues as well, vet them down south or wherever take them from and think that’s good enough. We do not. We’ve come to realize that some vet clinics in these intense areas, whether they don’t have... depending on whether were talking about Mexico or LA or whatever, whether their education is different or just the mass volume that they see because of the need, we feel very important to re-vet them here. So we do that as well. So cost before they come, transportation, medical, sometimes you pay a pull fee, it all depends on that rescue is doing, and where they’re getting them from, and what the agreement is.

**Follow-up:** Thank you. You’ve already mentioned this slightly about donations, but how do you recuperate these costs?

**Participant 6:** Social media is our major fundraising… uh now with COVID especially we haven’t done events, and I again I’ve always been hands off with events, I’m going to start now that I’m in Victoria by doing a bit more, but online stories of the dogs, and for donations. And honestly, most of our donators are people that I personally know. So, it’s kind of like how I knew them along the way.. again I have the investment banking background so I’m pretty good with… and [Name] is an accountant, so the two that are running the organization are very good at budgeting. So there’s the shortest answer. But yes we will have donators, fundraising, and as we’ve grown that has gotten easier.

**Follow-up:** Right. And has there ever been a time you couldn’t rescue a dog because it was too expensive?

**Participant 6:** Yes. Yeah. Well, it’s just a judgement call. Like when we first started out, we would always pay, every medical bill from LA. And some, we didn’t have the funds for. And when we first started out I had a dog in care I did rescue from the LA area, he was amazing, he became, about a month after getting him, I guess he had a slipped disc or whatnot and became paralyzed. So I drove him down, I was in Squamish at the time, saw my Squamish Vet, we drove down to... I can’t remember what they’re called in Vancouver… one of the big hospitals, it’s all specialists there and they basically said he needs a surgery, 50% chance and it’s $7,000. At that time we didn’t have the funds. And he was an older dog, and we waited. And we decided on the vet’s advice to put him down. But it weighs on me every day. Because we’re now… Fast forward to now, we’ve just spent $7,000 on a similar kind of dog, of age and so on, on hip surgery, and he does have behavioural issues whereas the other one didn’t. So anyways, it plays a part on my conscious of where we’ve come, what the budget is. But there’s been times in the past where we either had to turn down or not been able to do emergency risky situations. Again, if it was 100% I would’ve spent the $7,000. It was 50% so…

**Question 4:** Tell me about some challenges you face through your work?

**Participant 6:** Yeah, I mean if we have the time, haha. Umm, okay, I’m going to try to categorize. A major challenge is, again and I’ve kind of expressed with is, it’s high burnout right? Compassion fatigue, I didn’t know what that was until I developed… Rescue, has made me develop anxiety. My Facebook feed, I’ve had to unfollow a lot of things, weed things I saw posted through various rescue groups. It’s horrendous, what you see goes on. [Torture] houses of dogs, dogs with firecrackers tied to their mouth and… you know have gone off. Tons of various things. So, you know, it would anger me in my life because I would see my family continuing on with thanksgiving and doing these things and I’m here on my phone seeing these horrific things. And then you kind of feel alone because they’re like “oh you got too many dogs in your house”, and they’re not seeing what you see, so you kind of alienate yourself. So that was a learning curve that I had to… And that hits you in, year four. It kind of, you have to learn to shut off and then I feel like a hypocrite because I’m shutting off like everybody else does… Anyways, so, challenges. Yep, there’s a mental challenge.

There’s frustration in that… you know, we’ve worked so hard to educate ourselves in humane treatment of animals and people who quote on quote… like now me as a dog trainer like, seeing a dog even on a shock collar walking by enrages me. And I have to keep my mouth shut and keep my husband and everybody happy so I’m not attacking everybody on the street, haha. So, there’s a personal challenge that I could go on and on about in the rescue itself. I mean, my team, they work so hard, this is 100% volunteer. They do it for the love of dogs, and people are horrible sometimes. Especially with COVID, there was a, we’re a small organization. We had 100 applications for one dog. So you have to let down 99 people and someone in those 99 are mad, that they’re not chosen. So, there is an array of challenges. Not having enough money to do things. My biggest challenge is people. And I’ve had to separate myself from a lot of it because, yeah. And people want to relinquish, so… another reason locally, we were going to start opening up to it but that’s a discussion we’re having in the next meeting but why we don’t local owner surrenders; it’s too emotionally difficult on me and to restrain myself as well to tell them what I think. And so, that’s why I had to for my own sanity write up.. So a lot of people go “ohh why don’t you rescue more locally?”. Again, my main points are, walk through that shelter, walk through ours. Also that I don’t want to deal with direct contact with owner surrender.

**Follow-up:** What can be done to reduce or possibly even eliminate these challenges that you face?

**Participant 6:** Um… I struggle with eliminating the… And another challenge sorry would be other rescues that ruin the name of rescues, right? Just like dog training, rescue, is unregulated. So, you have a wide spectrum of how people are operating in rescue, in dog training. We like to hold ourselves to the higher standards and do the best we can for the dog, our reputational risk everything. Again, we’re run by an accountant and an ex-investment adviser, so we’re very critical with how we work. And it’s also a challenge when a rescue, I can give one in Calgary, I’m trying to think of their name… oof, they got a lot of bad publicity recently. It will come to me… Anyways, when they... and again, I think these people come from well-meaning, but they get greedy or, numbers are more important than the individual, so I think those organizations present challenges to us because we are constantly being compared.

**Follow-up:** And just to clarify, are these rescues that you are referring to, rescues that also import dogs from abroad?

**Participant 6:** Yes.

**Follow-up:** Right. So, would having more structure, or control over being a rescue help with this issue?

**Participant 6:** Yeah… And that is hard to…. control. I think… Umm, yeah. I guess regulations somehow would help. Education, as well.

**Follow-up:** Education targeted at the rescues?

**Participant 6:** Again, now my mind works in a weird crazy way, but again if we can’t even regulate how the average Canadian trains their dog, I don’t know… Haha sorry. I guess in rescue it would be easier because you can implement stricter… but yeah I think it starts in a much bigger place for me as well.

**Preamble:** From what you’ve described during this interview, it must be very challenging and demanding to work in a rescue organization.

**Question 5:** Can you please tell me what it is that keeps you continuing your work?

**Participant 6:** The dogs. Yep. For instance, yesterday, and if you go on our Facebook page, we took in two dogs. And I had the honor of... Both bully mixes, which are passion for me to assist. I had to kind of step down from fostering in the last two years, because I had little human babies so, you know I have two dogs, two kids, it’s not a safe environment to bring in unknown dogs and also I had enough of cleaning pee and poo. So, I’m not able to foster at the moment and I really miss it and I got to go assist these two dogs and again, these are lovely dogs, one’s extremely geriatric, I look at him and oh my god, he’s on his last legs, but for him now to come into a home and his final years or months or weeks, or whatever, it is extremely rewarding to see that he has that opportunity. And the other dog who has a broken leg and healed that way, she has aa wonderful personality and just, yeah. Just to be able to give these dogs a second chance is so rewarding. And that’s what keeps us going.

**Follow-up:** Thank you, and congratulations on your human babies.

**Participant 6:** Thank you.

**Follow-up:** What do you enjoy most in your work?

**Participant 6:** Giving these dogs a second chance. Yep. And then also, you know, in regards of when I personally foster as well, I would take in harder shut-down cases and knowing that… again, my intentions were amazing when I first started but I didn’t necessarily have the knowledge base to help them through some of the behavioural issues. Now I have that, and so... Like before my daughter was born, I just went through and saved as much pets as I could and worked on them behaviourally because I have the background and that was extremely rewarding. I miss fostering. That is something that I really enjoy.

**Follow-up:** Right, thank you. In the perfect world, what would the lives of all dogs look like for you?

**Participant 6:** Wow... The big question. Um… The average dog lover is not dog educated. So again, I would like to see people learn about force-free training, learn about dog body language and what your dog is saying, and making that apriority. And so in the perfect world for dogs, these quote on quote “dog lovers” are dog educated. So, they treat dogs appropriately

**Preamble:** I’ve asked you a lot of questions today, and I want to thank you once again for sharing your thoughts and experiences with me.

**Question 6:** Before I conclude this interview, I’m wondering if there’s anything I haven’t asked you about that you think it is important for me to know?

**Participant 6:** Just again, it’s shocking to me when we feel that were under a bit of a microscope when we try to do the best we can but there’s a lot of rescues that re operating out there that aren’t.

**Follow-up:** Thank you. Did you have anything else you wanted to share? If you also have questions for me, I’d be happy to answer as well.

**Participant 6:** No, I think that’s great, other than, your study is then, finding the motivations, what is this to be used for?

**Follow-up:** Yeah, that’s. Great question. In my study… Or, from the start of my Master’s I was looking at dog importation, and in my first study, I looked at whether dogs from abroad have poor behaviour. So I did a survey to dog owners in Canada asking whether they saw problem behaviours, and other attitudes. And through that study I found that there were no increased dog behaviours problem for dogs coming from broad. And that led me to wanting to understand why there is even a discussion for dog importation in general. So I noticed that there’s two sides, there’s one side that says “we shouldn’t be importing dogs, there’s plenty of dogs locally, they have bad behaviour.” And there’s the other side that says “no there aren’t an overpopulation of dogs locally and there’s a need for the importation of dogs”. So I wanted to understand more on that I’m interviewing rescue organizations. And by getting their voices out, and their concerns and challenges, I’m aiming to inform the general people that are partaking in this debate.

**Participant 6:** Okay, awesome. I love that. So the behaviours, interestingly enough… So I’m originally from [area] and last few years I was in [area]. I was not a trainer while living in British Columbia, again I was in investment banking and then in Calgary became a trainer. I saw, very interestingly enough, in my clients, so I did start a dog training business. Again, one of the points you had was, is there difference in dog behaviour abroad versus locally. I do think sure, there’s going to be some areas, even in Canada, there’s many areas in which you’re seeing different dog behaviours, minutely. Because for instance in Calgary a lot of my clients that was in this canine class I held which was for shy reactive dogs to other people, a high percentage of them were born in farms in barns. So again, you weren’t getting that critical period of socialization. So, you’re not getting that as a pup and here they are, Canadian dogs. So yes, geography and lifestyle of where that dog came from is important, but like you said, it’s not necessarily an importing issue. It’s just as socialization period issue. Some issues that there is with importation is people are importing and don’t understand the diseases of where you’re taking from. So that I think, yeah. Getting stricter on that. And again, well-meaning people, but just lack of knowledge. And I’ll say small dogs are.. Even Manitoba, you look at their need. They area importing small dogs to Manitoba. So if you look at Canada as a whole.. And we, the problem with us is that I don’t like to be a small dog rescue. Again, pits have a high need in the LA area. My god, you walk through a shelter there, 90% of the dogs are pits. Depending, but the area I’m talking about. But, so when people talk about the debate of import or not import, yes, Canada has a need. Even where I rescue from in Canada, very little small dogs. So we have a shortage of small dogs for the need. And one thing I would be curious, and I don’t have the time to research so an idea for you.. and again the rescues that I think might be bringing down some of the… There’s a lot of rescues that you adopt from internationally, right? So you go directly and.. they have someone who works in Canada and helps them, but there’s not footing in here in Canada. So, you basically apply for a dog online from China, from Texas, from wherever, and then you get a dog. My question is, what happens when these don’t… and I have a best friend she adopted from Texas. I think somebody from Kamloops or something helped out. But again, what if it didn’t work out. Where does that dog go?

**Follow-up:** And is that a rescue?

**Participant 6:** Yeah. There is an oversea rescues that basically do direct adoptions do Canadians. You go online, you go apply for the dog, and they fly the dog to you directly. And you go pick up the dog. To me, that’s fine and dandy until there is a problem.

**Follow-up:** I see. And I could see how that goes back to your point of getting that, getting grouped as “rescue” in general

**Participant 6:** Exactly. So, we’re getting grouped with them. So, for instance, I’m newly back to [location], but the SPCA then gets these dogs relinquished to them that have no rescue backing them. So now they are kind of you know, kind of got their nose up to these private rescues. Again, these are the ones causing issues because their dog is ending up in the shelters with no rescue to back them. So, there’s an issue in importation.

**Follow-up:** Thank you for sharing that. As I do my, research I’ve noticed that there’s so many different parts to even just the “rescue”, so I would love to just even mention these parts and highlight so many different things that are happening.

**Participant 6:** Exactly. Because there is no quote on quote “governing body”. So, like you said we’re being grouped. And if rescues taught me one thing, and I catch myself still, because I came from very judgmental parents, but don’t judge until you fully know. And again, I don’t want to completely judge yet, that they’re bad rescues. I don’t understand what their plan is when it doesn’t work out. There may be an answer that is legitimate, I’m just not aware of it. So, it’s like “don’t fully judge and make an opinion until you have all of the answers”. So, anyways thank you for doing this.

**P7 Interview Transcript**

**Introductory question:** Can you please remind me, from which geographic areas does your rescue organization import dogs?

**Participant 7:** Mostly from South Korea. And I’ve just brought my first from China.

**Question 1:** Can you tell me about how your organization came to the decision to rescue dogs from these areas?

**Participant 7:** It’s a little bit of a story. Is that okay or do you want just an answer?

**Follow-up:** A story would be great.

**Participant 7:** Okay, so I was a flight attendant with [Airlines company] and one of my (South) Korean friends goes back and forth, has family and a home in Korea and at one point she showed a picture online of a little dog, and said it was in her neighbour’s yard, tied up on a chain, it was a puppy and her mother was also tied up on a chain, and their whole life was on this little cement yard, and did anybody want to adopt it. So I was going away so I thought I can’t but I shared it. So when we came back from that vacation, my husband and I decided it was time for us to get our dogs again, and we were going to try and get a bonded pair and then my friend said “what about that [Name]’s little dog she shared? Maybe that’s still available?”. So, I emailed her and she happened to be in Korea and we had our little dog come over the next Tuesday. She was a ball of energy and so we decided we needed another dog to keep her occupied. And we ended up adopting from [Animal Rescue] in Squamish. And ended up with a dog that looks sort of Benji-like and found out that it had come from Korea through California, was adopted in California, was returned in California, and ended up in Squamish, and we got her. And after doing our research, we discovered she’s part Korean, sub-Sahari, which is a highly valued breed in Korea and bred by a special location. You can only get them... like they’re tattooed and stuff. Except she’s a mix. And my friend [Name] who got the original one was at home in Korea again and she checks the pound again everything and sent me a picture of this other sub-Sahari mix who was very different looking, sort of scruffy looking. Fell in love with him. And I said, we’ve got to bring him over and find him a home. And that’s how I got in touch with the [International Shelter] who I rescue from, and I would pick and choose a few dogs, the ones that were different looking that don’t get… You know they like the Jindo’s there and they like small dogs, so I would pick the setters, and the different dogs the ones that look scruffy and different and black dogs, and I would bring them over. And then they had their… euthanasia list came out last spring and that’s when I really got into and worked hard and got a lot of dogs adopted and we still have dogs waiting to come over because they need the flight volunteers but that’s basically how I started it and the rest is history. It’s been just a year now.

**Follow-up:** So just to clarify, does that mean that your rescue organization was focused on foreign adoptions, and rescuing dogs from foreign countries?

**Participant 7:** Yes.

**Question 2:** Can you tell me about the factors you consider when selecting dogs to bring over internationally?

**Participant 7:** For me, it’s health and personality. I don’t have... It’s just me so, like my husband says “as long as it doesn’t cost us money”. And so, I can’t.. you know, look having a lot of fosters and stuff like that where you have to pay for food and things like that, I can’t afford to do that. So one of my criteria when somebody contacts me if it’s anyone other than [International Shelter], I say “I can’t take anyone with aggressiveness, I don’t want aggressive dogs.” We don’t need to bring more aggressive dogs, they have lots of dogs in the pounds, here they have a lot of big pit-bull type dogs that, the ones that don’t get adopted here. So, that’s a priority for me but apart from that, the look, I’ll take the weird looking ones I like the weird looking ones. The ones that are not going to be adopted over there.

**Follow-up:** Right. I think I’m already slightly getting the answer to this next question, but can you describe to me what a “typical” dog that you rescue might look like?

**Participant 7:** Well, I’m drawn to the Sub-Sahari, sort of terrier, scruffy look. I’ve got waiting… I’ve got a little, I don’t know what he is, he’s white and got bendy legs and he’s really tough little guy. He’s waiting to come over. I have an English setter type dog that’s waiting to come over. I brought over another hunting dog that… What I did, when it’s a specific breed like that, I will contact the specific breed rescue here and sometimes they will help me. And through that one I found an adopter for this one particular dog and he is just living the life now. He is a hunting dog but that’s what she was looking specifically for that breed and she waited while he went through heartworm treatment, and everything and he arrived over a couple of months ago and he’s doing just amazing.

**Participant 7:** Thank you. What is life like for a dog before being rescued?

**Follow-up:** The dogs that are at the [International shelter], which is what I mostly rescue with, they usually come off the road. Some of them are from the meat trade, but a lot of them seem to be picked up abandoned dogs up in the hills or in the city. Like one of them was tied up in an abandoned building, the one that came over recently was found in the forest, near the forest. Some of them the mother was brought in and they were born at the pound. It’s a real mish-mash at the [International Shelter]. It’s government-owned, so they do get medical care and stuff like that. So that’s the good part for me, dealing with them is that they do get better care than maybe what some other rescues might. But they come from all over. It’s mostly abandoned animals. I think what happens in South Korea is, they don’t have a pet culture as much as we do here. So, they get a cute little puppy and they don’t understand the training and bringing it up so it’s a well-behaved dog. So when it gets bigger and they live in a smaller apartment, so it’s like “oh I can’t.. This dog is crazy, I’ll have to get another one”, so they just abandon it. So that happens a lot.

**Follow-up:** Would you say then, that it’s also related to the breed, that they get physically too big, or is it more so to do with their energy level?

**Participant 7:** Um a lot of them get big physically. Like, I know that there is another rescue that brings in a lot of golden retrievers who are very cute puppies but, oh my god have you ever met a full-grown puppy golden retriever, like a 1-year-old? Like, their energy, if you don’t train them, they’ll jump and pull and, I mean can you imagine in a small apartment? It’s just unmanageable.

**Follow-up:** From talking to other rescue organizations, they often mention that they some procedures to find the “right match” between the dog and the owner. Does your rescue also have that, and can you please describe that looks like?

**Participant 7:** So what happens um… I do the interviews for [International Shelter]. So people apply for [International Shelter] for a particular dog. And then they’ll send me the application form and I’ll set up a zoom meeting with the people to interview them. I’ll find out about their lifestyle; I always ask them “tell me, a day in the life if you have a dog how is it going to change? What are you going to do as far as walking, exercise, training?” And I just find out… I also get a vibe from them, you know, if somebody’s very nervous then I’ll go back to [International Shelter] and I’ll write my review and I say “well, she seemed very nervous so tell me more about the dog that she’s applying for?”. Because we’re a little bit different in that, the dog is adopted and then it comes over, generally speaking. I don’t do a lot of fosters because, as I’ve mentioned before, I just can’t afford… It’s a lot of work having fosters. Plus, I get very attached to the dogs and I get really, really fussy about where they are going. So, when you get to know the dog personally it’s a little bit harder to place them, you know. You want to make sure you’re doing the best for the dog.

**Follow-up:** So just to clarify, these people that apply for the dogs, they would be people in Canada, and they apply to the pound directly?

**Participant 7:** No, I interview for people in Canada and I also do their interviews for the east coast for the United States for them. And because they don’t have any rescue partners on that side; they have somebody in the west coast, but not in the east coast. So I do the interviews, and then they arrange the flight volunteers and the dogs arrive.

**Follow-up:** Right, and through the interviews, you get a sense of the adopters, and match that with the dog’s bio?

**Participant 7:** Yes, generally speaking, as far as [International Shelter], the people apply for a dog, and then they will go back and forth with them and try and find either a really good match for them or if the people want a specific dog, then they’ll go over and make sure it’s matching and they’ll give me that information and I’ll do my interview, I’ll give my feedback, and I’ll you know, I’ll follow up on financially… If it’s young people, I always make sure that they understand that the cost of having a dog is horrendous and can put you under very quickly if you don’t get pet insurance. So, if they’re young, just starting out, they got their first apartment, they got a job and now they feel like they can afford a dog, I tell them how much I spend on vet bills in a month and they’re like “oh, okay”. I say, “you need to get pet insurance”, you know. That’s almost a requirement as far as I’m concerned.

**Question 3:** What are the costs that are involved with rescuing dogs from abroad?

**Participant 7:** For me?

**Follow-up:** Yes, from the perspective of a rescue organization.

**Participant 7:** For me, it’s not too bad. I live in [Location]. So, if the dogs are coming into the airport, I will take the ferry over and that cost, the ferry cost is added over to the adoption fee. If I don’t need to go over, then it’s just the adoption fee. And the adoption fee is purely, it goes back to Korea. I’m a volunteer I don’t make anything on it, I just cover my costs, if I have any costs. Like right now, I have the two dogs that came from China and I’m taking them to the vets so I’m trying to do a fundraiser to cover the cost of the vetting and I have, you know, regular people who have adopted through us who will always give a little bit and help with the expenses but, really it doesn’t cost that much if I keep aware that… when they arrive they’re usually adopted so they go straight to the adopter so I don’t have costs there. Umm, I don’t know if I’m answering your question. Or are you asking, specifically the cost of bringing a dog over?

**Follow up:** Usually, from a rescue perspective, there’s medical fees associated with a dog, certain flight fees but I guess in your case it’s a bit of a unique case.

**Participant 7:** Yes. So the adopters pay the cost of the flight. In Korea they cover the medical, and everything is all covered. The adopters may for the crate, flight, the expenses like, they have to go into quarantine the day before so transport to the airport if they don’t have the volunteer to take the dog to the airport. So, generally speaking it’s about $750. That’s a very average price. We do have people who pay cargo for having their dogs brought over because they want them sooner and its astronomical. It’s a sin what they’re charging. It really is not fair. And it’s gone up through the roof since covid.

**Follow-up:** Has there ever been a time where a dog was too expensive to bring over?

**Participant 7:** Well, I mean it’s so expensive if you have to… Let’s see. I have one dog in Korea who I will bring over hopefully eventually. And we’ve done fund raising for him. He’s got quite a following but he’s very broken, very shut-down. And so he’s in training and that has costed quite a bit. I did fundraising on my end and they’ve done fundraising on their end. But he’s a very shut down dog, and would have to be a very specific person to adopt a dog like that. So that can cost a lot of money, but generally speaking it doesn’t cost me to bring them over. The first dog I brought over I did pay for his cost to come over, but then the adoption fee I got covered that, so it was just a wash.

**Question 4:** Tell me about some challenges you face through your work?

**Participant 7:** Yes. I can. We’ve had one adoption that failed. Went to Boston. It was just a disaster. This girl had no clue how to approach this dog. A shy friendly dog when it was in Korea and it just... I guess it growled at her and she just freaked out. So, we had to hire a driver to drive from Ontario to Boston to pick her up. We had to call animal control to remove the dog from her and hold the dog for us. We had to find a foster, pay the expenses of the foster. So, we had to do major fund raising for that. She asked for the refund for the cost of her dog, and it was going to cost a lot of money and I said “Actually, you signed on your contract that if you had to rehome the dog, it’s at your cost. We’re just going to charge you the same that it costed to send the dog over.” I just sort of just sent it and crossed my fingers hoping that she would agree with it, because that’s what she had agreed to. And then we just fundraised for the rest of the cost. That dog is still in foster, a wonderful foster home. Still learning, it actually bit the husband. Not aggressively but reactively. They’ve been wonderful, but now I’m trying to find a family for that dog. And that’s a little bit more difficult when you have a dog with an issue like that. That’s the only failure that we’ve had. We’ve had others that came over and they thought they’re going to have a friendly happy dog and they have an aloof dog, it’s the personality of the dog doesn’t want to be with other dogs. But they’ve worked with them and they’re so wonderful with their dog and they give their dog all the time and patience they have. But it’s when a dog arrives and it’s not what the owner, the adopter thought they were going to be. Like a really friendly dog, it could be very shy. And it takes a while, I’m sure you’ve heard people talk about “3 days, 3 weeks, 3 months” rule. Personally 3 days is not enough. I think it’s minimum 2 weeks to see the personality come out. And then it progresses from there. I have two big labs that just came from China, and I’m trying to find a home together, they’re bonded. I may have to make a heartbreaking decision to split them up, but I would like to get them on the (Vancouver) islands so they could at least have play-dates. But that was just a leap of faith that I took to get them over there. They’re very sweet dogs. But, when I’m walking them on leash, they’re very reactive when they see another dog. Like barking. It’s not aggressively but, its sounds aggressive. But, you know, I know they’re okay with other dogs from the pictures I’ve seen before. But you never know what you’re going to get, you just don’t know what you’re going to get. You have a lovely dog over there and you put it into a different situation here and, you know. We explain that to the adopters too.

**Follow-up:** Yes, it is a completely different environment for the dog, so.

**Participant 7:** It is. And one of the things that’s interesting is “Oh, I’ll take it for lots of walks and everything”. Don’t do that, they could’ve been in the pound for two years in a small cage, they don’t have the muscle mass. You’ve got to think about where the dog is coming from. Also, it’s very quiet in your home compared to living in a pound with all the dogs barking. So, they hear all sorts of noises that will stress them that they’re not used to hearing. So, you have to do your education. We stay in touch with our adopters. We have Facebook chat groups with all of our adopters. And they can ask questions anytime. We never close the groups.

**Follow-up:** You mentioned that one of the main challenges that you have is when the adopter and the dog doesn’t have this “good match” that you were hoping that they would have. Is there anything that could be done to reduce or possibly even eliminate these challenges that you face?

**Participant 7:** The Facebook chat groups are critical. We have myself, the adopters, and the team in Korea, who are people who work at pound, volunteers from the pound, the volunteer walkers who have lots of information about the dog. If something goes wrong that they can say, “oh you know, she never did that here so work with this”, and it’s just patience. It’s having patience and just being supportive with them and understanding that, you know. It might not be what they wanted but they’re not going to give up on the dog, they’ve all been really good with them. But, generally speaking we’ve done pretty well. I’d say our record for having done this for a year now and only having that one dog who just was a failure.

**Preamble:** From what you’ve described during this interview, it must be very challenging and demanding to work in a rescue organization.

**Question 5:** Can you please tell me what it is that keeps you continuing your work?

**Participant 7:** Those little furry faces. Just seeing them and… I think because of COVID I’ve put more time into it than I really intended to, and I’m sort of.. .When we did the euthanasia list, that’s when it really exploded. So now I’m just kind of, started pulling back, not getting as much. Because here in Vancouver, because I did so much work, we have a long line-up waiting for flight volunteers. Like in Toronto, I have two dogs waiting for flight volunteers, here, I have 25 dogs waiting. So, I’m actively trying to find volunteers all the time. My friend [name], is wonderful. Any of her friends she hears is traveling, she’s like “you’re taking a dog”. So, that is probably our biggest challenging, is finding flight volunteers.

**Follow-up:** Is that also specific due to the COVID-19 pandemic?

**Participant 7:** I think it’s probably a bit harder due to covid, but I mean, the flights are full though, so people are coming. Last year I think it was harder, it definitely picked up within the last few months but I know that in Korea now they’re shutting down a little bit more and being more restrictive again.

**Follow-up:** That’s very interesting. So, people are travelling but they don’t want that additional stress of bringing the dog with them?

**Participant 7:** Well, there’s no stress involved for them. They meet us at check-in, we have done all the paper-work, everything is done. We check the dog in, and they pick it up when they arrive and bring it through customs where they are waiting to pay all the bills or, you know customs fees and stuff like that. We have money for the border, they just have to pick the dog up and have a porter bring it out to us. If they’re bringing a dog on board with them, a small dog, then they carry the dog with them and that’s it.

**Follow-up:** Sorry, this might be a tough question but why is it then that flight volunteers don’t want to volunteer?

**Participant 7:** I guess it’s finding them. Like letting them know and getting the word out there. They’re not aware that they’re needed. Also, with [Airlines A] for example, there’s only one dog allowed in the haul because of the aircraft type. So you can have one passenger bring one dog. And you can bring I think, six in the cabin, maximum. So, six passengers with six dogs. But, most of our dogs are a little bit bigger than cabin size, so, for [Airlines A], that’s the problem. I think [Airlines B], we can take more, I think there’s four or five, something like that. And it’s to do with aircraft type. [Airlines B] has temperature-controlled units for the dogs, whereas [Airlines A] doesn’t. So [Airlines A] will also have shut-off periods where it’s too cold for the dogs to travel under certain temperature and too hot in the summer. So it reduces that window of opportunity as well.

**Follow-up:** Thank you. Going back to why you do your work, can you explain to me what it is that you enjoy most in your work?

**Participant 7:** Handing the dogs over to the owners. That’s always fun. And getting feedback.. I don’t know if you’ve been on my Facebook page but we do [regular updates for dogs], and when they send me pictures and I put them on there and, my followers like that too. And sometimes we have two or three dogs arrive together so we had a chat group and those people know each other so they follow each other’s stories as well. So it’s definitely staying in touch with them and hearing like, we had one little.. One family who wanted.. I don’t know if I’m running out of time for you..

**Follow-up:** Not at all we have plenty of time.

**Participant 7:** When I first started I had a little… A little dog. And they’re very popular, people want little dogs, they’re hard to get here. So I posted it and I had this email from this lovely lady, she was older so I did the typical… what a lot of rescues will say, “what happens if something happens to her, who’s going to get the dog?”, and I sort of left it at that. So I wrote back to her and said “I’m sorry but we’ve selected the owner, and unfortunately you didn’t make the cut this time”. She wrote back such a nice letter “I’m so thankful, I’ve written to hundreds of applications and you’re the first one that has responded to me”. And, you know, people are busy but I was just starting so I had time to do that. And I said, “oh I’m so sorry. You know the thing is, for an older person and you know, if something happened to you, what would happen to the dog?”. And she’s like “Oh my daughter lives literally across the street in the same complex.” And I said “Oh, I wish I had known that!”. And so I learned, always to ask that question when older people apply. So, they couldn’t get that little dog but they selected another one. This little dog arrived over and turned out it has vision problems they hadn’t noticed at the pound. This dog is the most loved dog you have ever met in your whole life. It has brought so much joy to this older woman and to their extended family, and to her extended family. They’re one of my biggest supporters, they’re always saying “we’re so grateful for this little dog. He’s just brought so much joy to our lives”. That’s what it’s all about for me. This little dog was in a cage in Korea, and had vision problems, and now it is here and adored. That’s what keeps me going.

**Follow-up:** In the perfect world, what would the lives of all dogs look like for you?

**Participant 7:** They’d all live somewhere like Salt Spring (Salt Spring Island) where they can run off-leash and have yards and a full belly and a soft bed. That would be… And you know what, they don’t even have to have that like, we have dogs that have been adopted by younger people who are in apartments who have amazing lives. Get lots of walks every day. When you think where they came from, it’s very hard to say no to anyone based on their apartment size or something like that. I’ve only done that once, I had a lovely couple, a young couple who wanted to adopt a second dog, they had a dog. And I did my interview, I thought “ah these are wonderful, they’re really nice people that’ll be great”. And I said “can you just show me your apartment?” And they showed me their apartment and it was really small and really cluttered, and sadly said to the [International shelter] people “you know, their apartment is just too small. They have a dog, they want another dog, they have a cat, they had a fish tank and..” lovely people though. And they emailed me afterwards and said “can you tell us, like we really thought we were doing okay”, I said “honestly, it was the size of your apartment. If you ever move, I’d be happy to let you have another dog if you have a bigger place, but we really just felt it was so tight in there”. And they said “I totally understand, that’s fine, thank you”.

**Follow-up:** Thank you for sharing that. I can see that you’re quite in-touch with the owners.

**Participant 7:** I think that’s something that’s different for us too. I know that… where we adopted Mocha from, I’m in touch with her and she’s helped me as a rescue, she said “careful what you get into”. Haha, I didn’t listen to her. But she doesn’t contact me, she doesn’t have a Facebook group for me. Ours is very much more personal. And that’s how I like to do it. I like to stay in touch.

**Follow-up**: Thank you. I’ve gone through most of the questions I’ve prepared for the interview, but I realized that I forgot to ask one question so I’m going back in reverse of what I had planned.

**Participant 7:** Okay

**Follow-up:** So now that your rescue organization is established, when you establish new connections with rescue organizations in other countries, do you usually have to reach out to them? Or do you get contacted?

**Participant 7:** The rescues from other countries have contacted me because of my profile is getting bigger I guess. I’ve just been contacted from one from Turkey, who I will work with. And there’s a reason why I’m agreeing to do that. There’s two rescues that have contacted me. The one from Turkey, she’s based in the US but brings them in from Turkey but they pay the flights. So they just want to get the dogs out. So I can charge the adoption fee, And the adoption fee then I can use for my fostering expenses and stuff like that. So, it’s a symbiotic relationship that I can help them get the dogs in, and I will make a little bit of money that I can use for my dogs. And there’s another rescue in (South) Korea who has asked me to do the same thing.

**Follow-up:** Wow, thank you for elaborating on that. So is my understanding correct that, when you first started, you already had this relationship with a rescue worker or a rescue, but now that your rescue is established, you get connections without you having to reach out to them.

**Participant 7:** Yeah, I actually don’t want to reach out to anybody else, I’m busy enough. And because I’m [at location], if I have to go over to the airport to pick the dogs up, that’s a whole day. I get up at 4 in the morning and I get home at about 11 o-clock at night, just given the ferry situation. So, it’s a long day and I’m not getting younger, so, I mean, I still do it. I will do it. There’s a girl… there’s a couple of rescues that I work with. There’s a girl in Toronto who has a rescue who did very similar stuff to me. And we help each other out because she’s in Toronto. She got me the foster for the failed adoption. And I’m in BC so I try to help her with the dogs that come into BC from her adoption.

**Preamble:** I’ve asked you a lot of questions today, and I want to thank you once again for sharing your thoughts and experiences with me.

**Question 6:** Before I conclude this interview, I’m wondering if there’s anything I haven’t asked you about that you think it is important for me to know?

**Participant 7:** Umm, I think one thing that’s really interesting, and you may have noticed this yourself but it’s mostly women who do this. Um, I know of one or two men… In China there’s a man who runs the rescue there, he’s from India. But everybody else, almost exclusively that I work with, that I communicate with, is female. So, it’s very much a female dominated, for want of another word, industry. We communicate, we help each other, like the one that put you in touch with me, they have helped me enormously. The one that I got Mocha from, they helped me initially with paperwork, what do I have to do. I mean, I had no clue. I hadn’t a clue about anything. Probably the biggest challenge with me is dealing with customs. Because it’s who you get, and if they had their coffee that morning. Honestly, some... I’m getting better now but when I first started because I was a flight attendant, so customs, you respect what they need, you do what they need. And I would try and… I went in the first time to do the paperwork and you have to do it on a computer, and you have to put it in a very specific order, clip it together with a piece of yellow paper, and I’d give it to them and they’d say, “it’s not in the right order”. I’m like, “seriously dude, you can’t move the order?”. Like there’s three pieces of paper, and I had them in the wrong order. They’re that unhelpful. So, I would say customs is probably, was. It’s getting better because I’m getting better at it now. But that was probably one of the biggest stresses. I said, “if I ever quit rescue, it’ll be because of customs”. Yeah.

**Follow-up:** Other rescues has pointed out challenges with customs as well.

**Participant 7:** Yep. And you know where the easiest place I just discovered we did it, was handing them off at [location] in Surrey. Right down by [local area]. Where the driver will bring them from Seattle up to the border, and they just, literally at the edge of the road, they hand them off and then you go to customs and do the paperwork. They were so nice there they didn’t even charge fees. So, I was like “I need to do this more often”.

**Follow-up:** Wow really? Less complicated than picking them up from Vancouver Airport?

**Participant 7:** Yeah the most complicated ones are the ones that come in Cargo. And the ones at the airport are usually pretty good. They’re not too bad. But definitely for me. I’m not a paper works person. I’m a hands-on, touchy feely, Libra, you know. I’m not an organized paper works person. So, for me, the paper works, and keeping everything straight, finances and stuff it’s the hardest for me. And that’s just my personality.

**Follow-up:** Great. Thank you again for sharing that with me. Is there anything else that you wanted to share before I conclude the interview?

**Participant 7:** I’d love to ask you a question.

**Follow-up:** Yes, of course.

**Participant 7:** What is... Like you’re… Animal welfare?

**Follow-up:** Yes.

**Participant 7:** What is your future? Like what are you going into with that degree? What will you be doing?

[Discussions from this point were not related to dog importation, thus was not transcribed.]

**P8 Interview Transcript**

**Introductory question:** Can you please remind me, from which geographic areas does your rescue organization import dogs?

**Participant 8:** That’s a loaded question! If I can remember, uh Mexico, Saint Lucia, I’ve done Virgin Islands, Columbia, Dominican, Costa Rica, Jamaica, Bahrain, Egypt, Nepal… I think that’s all of them.

**Follow-up:** Wow, that’s quite a list!

**Participant 8:** I mean the Nepalese one was a one-time deal, we’re probably not going to do it again because the cost is extremely high. It was just a specific situation so I’m not consistently working with these areas all the time. The ones I work with consistently are Dominican, Mexico, Bahrain, Egypt. Those ones we have more of a relationship. And I also work with Northern Ontario rescues, so locally as well.

**Question 1:** Right, thank you. Can you tell me about how your organization came to the decision to rescue dogs from these areas?

**Participant 8:** Well, before I was an organization and had a board of directors it was just myself, and I started with… How the heck did I start… I just started volunteering at different rescue type scenarios like fostering dogs for other rescues and I made some connections to ladies that rescued dogs from Mexico. And I had never pulled a dog from there and then a special case came up of a macheted dog that had spinal cord injuries and they don’t have the facilities there to deal with that, or cope with that level of injury so I asked if I could take the dog and they hesitantly gave her to me, and I got the treat… So it’s a long story. This is what I love talking about so I could go forever but anyways, I signed up to take on a very difficult dog that happened to come from Mexico and from then, I created a connection sand bonds and once that ball is rolling everybody starts asking, right?

**Follow-up:** Right. Thank you for sharing that. Also, I don’t mind if you go on really long tangents that’s really interesting for me to hear all these stories so that’s not a problem at all. Ok, so you initially got in touch with someone who was from Canada who was rescuing.

**Participant 8:** Yeah, I just started rescuing for like wildlife sanctuaries here, and I’m also a dog trainer. So a few of my clients who came in for training were rescues and somehow I made some friendships and decided I would foster some dogs. And I reached out to a couple organizations, and because I’m a trainer I became a highly valued foster parent, and then from there I just started rescuing on my own under my own name and then that just kind of snowballed into what it is now, and I have a board of directors and I don’t do any of my own admin anymore but I do, do the handling. So, yeah, it’s been many years of this. I’ve been in the dog world for 24 years.

**Follow-up:** Wow, that’s a really long time. That’s actually how old I am, so you’ve been involved in this for my entire life. I’m also interested in this “push/pull” dynamic of rescues. Now that you’re an established rescue, do you find that there’s a lot of requests that are coming your way to help, that you are kind of overwhelmed?

**Participant 8:** Yeah, we had to actually re-structure our protocol. So, we have a limit capacity that we stick to. We do get a lot of requests. Absolutely. From abroad, locally, in particular, we get a lot of behaviour requests locally, and then from abroad, the ones we feel that might do good here or need the care that they can’t get down there, we will take.

**Follow-up:** Ok, thank you.

**Question 2:** Can you tell me about the factors you consider when selecting dogs to bring over?

**Participant 8:** We don’t have a rule book. I think we just go by the seat of our pants as far as what we emotionally get attached to on the screen. I do have a group of specific people I work with, let’s say Mexico, and I personally love the medical cases like the spinal cord injury or the ones that are missing something. I find that they are highly discarded there, down in those areas of the world, yet very, very highly prized here so to give them that opportunity would be great. I’m fascinated by being able to provide the level of medical care that just is, completely unmatched sometimes in those areas. So, like spinal cord injuries in particular, we have hydrotherapy, and all these specialist doctors and surgeries is just unheard of. So, if I see something I think we can financially cope with, and the dog has a good personality then yeah. I don’t think we have a rule book for this. We definitely... It’s an unspoken standard, that the younger the cuter the dog, it’s going to “go quick” as we call it.

Behavioural issues are a big concern. I don’t like to take on behavioural issues, but I’m known for it so. I think… Because I have a very small board, it’s me and two others, we kind of just pop a photo up if something comes in our feed or somebody asks, and we vote on if we think this is a good choice to take on that dog so, we kind of are open ended in that fashion. I don’t say no to any specific thing. I’ve definitely personally… I have six dogs that are under my care that are… We call them “residents” because I have a farm and I have a house. And those dogs are all difficult. So, I just couldn’t see… I don’t want to euthanize them for behaviour but I keep them because they’re safe with me and they’ll have a great life. Yeah, I kind of take everything? I don’t know.

**Follow-up:** Can you describe to me a typical dog you rescue? This might be a difficult question. I’m just curious about the dog’s aspect. So, if there even is a “typical” dog that you rescue. You also mentioned that you rescue locally, so if there’s a difference between there.

**Participant 8:** Here, locally if I’m going to the GTA (Greater Toronto Area) area of Toronto you’ve got everything. You can get… I just did a purebred Doberman. Right? Where did that come from? It just depends on the area you’re pulling from. When you’re working from up north, so Northern Ontario of the reserves, Manitoba, those are typically husky crosses. Husky and Malinois crosses. A lot of them. They may have labs, they might have mastiff but predominantly you’ve got husky crosses. When you’re looking at Mexico, and Dominican, and Jamaica, they’re what you call “Pop cakes”. They’ve standardized street dogs into a... It’s literally started standardizing itself into what we call “Pop cake”. With the Egyptian and Bahraini dogs, they’re more saluki crosses. So I can’t say there’s a standard but we definitely notice patterns of breed types that come from different areas of the world. And because I rescue all over, I have everything you can imagine. It’s like a Heinz 57 of dogs.

**Follow-up:** Thank you for elaborating on that. This next question might also be a difficult question because you rescue from all over, but can you describe to be what life is like for a dog before it is rescued?

**Participant 8:** Again, depending on the region. And this is why rescuing abroad is a little bit different from rescuing locally. More often than not they’re either A) street dogs or B) Born into a shelter situation. Very often, minimal handling, no leash skills, there’s not… it’s a different perception of how dogs are to be treated and how they are kept in certain areas. So, a lot of “firsts” for the dog. That’s the average I would say. Anything from over the border is street, and not feral, but usually very subordinate and fearful. I think somebody should actually do a study on this because we’ve got a lot of fearful subordinate dogs and my theory is, that’s how they survive, by being aloof. And if you’re forward, you’re going to get hit by a car or hit with rocks so, we’ve got a lot of that. And, there’s unique situations where certain dogs are a certain way because of their circumstance. Not all of them are abused. Let’s just put it that way.

**Follow-up:** Thank you. From talking to other rescues, they have some procedures to find the ‘right match’ between the dog and the owner. Does your rescue also have that, and can you please describe that process to me?

**Participant 8:** Yep. We have a… I’m sure they’ve all done this, all kind off bounce off of each other. So we have an application form, home check, two reference checks of that reference check, then we kind of take into account what the dog is in need of. So I’m not going to disqualify somebody for not having a backyard. Do you know what I mean? So, we kind of take it on a case-by-case individual basis. But yeah, we have a selection process that way. And if were torn, we actually kind of go on a first come first served, not necessarily that you’re the first one that applied you’ll get it, but the first best application will go first. If we are torn, then we post among the board and vote. Kind of go through the fine-tuned comments and see what the best match will be.

**Follow-up:** Thank you.

**Question 3:** What are the costs that are involved with rescuing dogs from abroad?

**Participant 8:** For us, there’s a little bit on transport but not much. We do a lot of escorting, you know escort. You know what I mean. We find escorts to fly the dogs when they’re on vacation. A lot of good networking that way, people that travel often are repeat escorts. A little bit on transport, mostly on vetting. Most of all of it is on vetting. As far of supplies, we don’t.. I mean I can get pounds and pounds of dog food for free and all that kind of jazz. I also run a board in kennel, and a daycare, and I train so I have supplies coming out of my ears. I think vetting would be the most costly, and then, we obviously have an adoption fee that goes right back to the rescue as well.

**Follow-up:** I think you’ve already touched on this next question by mentioning “adoption fees” I’m assuming, but how do you recuperate these costs?

**Participant 8:** The adoption fees. And fundraising. We do fundraising. We’re completely not for profit. We don’t pay for ourselves, its completely voluntary, but we have a good network and yeah, people regularly will do, anything from a garage sale to a photo-shoots, and I think just being at this so long I have regular donators. And again, the adoption fees we recoup that way.

**Follow-up:** Has there ever been a time where you couldn’t rescue a dog from abroad because it was too expensive? And in your case, it could be locally as well. Can you describe why that was?

**Participant 8:** Hmm, no I don’t think I’ve ever... I mean... let me just think about that. I don’t think we’ve ever been broke. We’ve just slowly grown and grown so much that I’m like “okay, were at a cap”. And once we reach that cap, it’s just for psychologically I don’t want to be so big. I know all the local GTA rescues. We all discuss in circles and groups and some of these rescues are gigantic. They’re doing hundreds of dogs a month. I’m small scale so, you know I pick what I need, and we’ve reached our maximum so I think we have a little bit more control over that way. And that kind of keeps our finances in check as well because we’re always on the up. There’s never been a time that we’ve not had funding.

**Question 4:** I think this next question is a bit of a loaded question, I notice that people always pause when I ask this question, but can you tell me about some challenges you face through your work?

**Participant 8:** Oh boy. Yeah. Um.. People. Uh, I guess yeah, I think biggest one for all of us is interaction, communicating, particularly with adopters, random people. There’s this expectation that this is our job, that we must answer them, or you know, in a manner that they find…. I think they need to understand that this is completely voluntary. And yeah, there’s disappointment if they’re not chosen, and just dealing with that emotional rollercoaster of people. And then, difficulties, yeah when the dog is sick or has to be euthanized, that doesn’t happen often, but it happens. Um, I don’t think it’s difficult to raise money I don’t think it’s difficult to care for them. We have a lot of foster homes as well, so I don’t exclusively have all the dogs. I have some, but not all. I think that would be the most difficult is kind of navigating, because we have this extensive, you know, application process, you’re delving into private lives and then they, always want you… And I’m a trainer too so I get a lot of “I need help with this” and I have to do it for free so it’s a little exhausting. Everyone’s exhausted. Most people drop out of this. They do this for a bit and they leave, right?

**Follow-up:** That’s so interesting. You, being a trainer, I think you’re in a bit more of in a unique situation. But by people do you mean just the adopters, because I know that rescues interact with people from other countries, customs, people on the internet, and just all sorts of people so I’m curious which people that you’re referring to.

**Participant 8:** I mean adopters. And I do deal with other rescues. Other rescues when they have a behavioural issue will send to my farm where I can assess and train for them. But yeah, I’m strictly talking to adopters or wanna-be adopters. Those are the people that, can make it a little harder for us. I mean in general they’re usually quite pleasant. But sometimes we have hiccups and it’s extremely frustrated and you get burned out from that. And you know, we kind of have to slow down sometimes because were not mentally able to take it sometimes. But c’est la vie.

**Follow-up:** Is there anything that could be done to reduce or possibly even eliminate these challenges that you face?

**Participant 8:** You can’t change people… You know what, in running my own business, I can’t change people, I can’t change their reflex and responses. What I do do, is make sure our number stay small. Like this is something, we literally… I’ve been at it for so long we’ve been registered charity for at least five years I would think now. What I do is I try to get the board in a better head space. We try to let things slide, we try not to get so upset over things that are literally nothing. So, it’s more working on ourselves to be able to cope with the stresses that come with this. I mean it’s literally parallel to running a business, I have to deal with people all day, most of them I love, sometimes there’s hiccups. And I can’t change them, I can only change how I let that into either bother me, or not bother me. So, we have meetings where we decompress and tell each-other “don’t overthink it, it’s okay. That person’s upset.” Or “that person is needy. Let’s just vent”. We’ll go out to dinners or lunches together and we kind of have that bond as friends as well. We try to kind of formulate different kinds of… Sorry, I’m rambling… To create a cap. And that was healthy for us, because we were pulling everything and anything and it was becoming very stressful. So now we have a number that we have to stick to, and that’s kind of cooled everything down for us.

**Follow-up:** So, the number of dogs that you can rescue?

**Participant 8:** Yeah. That we have in our rescue at one time. It’s ten. That’s our cap because we’re so small. We could absolutely, financially, and in any other way we can go over that, but we just chose to do that because it was getting a little too much.

**Preamble:** From what you’ve described during this interview, it must be very challenging and demanding to work in a rescue organization.

**Question 5:** Can you please tell me what it is that keeps you continuing your work?

**Participant 8:** I’m good at what I do. I’ve thought of leaving many times. I’m good at what I do, and I’m good at handling. And there will always be a need for it. And because I run a business like this, it does help my business that I run a rescue. Although I ran the rescue before I had my business, you know, makes people see me in a different way as opposed to other kennels. And then the fact that I’m skilled with medical… It’s almost like the best surgeon in the world deciding he’s quitting to become a surfer, it’s like “why your skills are so good!” Right? So, it’s one of those kind of things. Like it would be very disappointing to multiple levels of people if I left. So, it’s become a routine for me. I’m not opposed to rescuing. Just not on a crazy scale. In a very calm way. Small numbers, take what I can.

**Follow-up:** Thank you. What do you enjoy most in your work.

**Participant 8:** Making breakthroughs with people and dogs. Making breakthroughs. Like I’ve recently done... For some reason I’ll take on a project and then become the go-to for that project. So, I recently did a couple of feral dogs from Korea, like meat trade dogs. And since then, everyone’s sending me their feral dogs and I’m videotaping them day one, and day two, and day three and then they’re walking and everyone’s talking like “wow!” So, that’s really rewarding. Or when I get the spinal cord injury dog to walk or get him a proper wheelchair. Those little victories. And then when they find a home, I’m really stoked about that. So that’s what makes it worth it.

**Follow-up:** In the perfect world, what would the lives of all dogs look like for you?

**Participant 8:** That is probably the most loaded question. I think everyone’s got their own standard of care. I think here were a little, coo-coo. A little too much. Shelter, water, love. I think they’re domesticated, right? We’ve created them, these aren’t wild animals, they’re domesticated dogs. So, they’re our problem now. So yeah, I would like... And seeing what I see when I go to Dominican or Mexico, I know what they’re like there. I would want them to be disease-free, I would want them to have indoor and outdoor life, and proper water, food, and care, and no abuse. That would be enough for me. Do they need angora sweaters and booties? No. So, I’m very simple that way.

**Preamble:** I’ve asked you a lot of questions today, and I want to thank you once again for sharing your thoughts and experiences with me.

**Question 6:** Before I conclude this interview, I’m wondering if there’s anything I haven’t asked you about that you think it is important for me to know?

**Participant 8:** I was thinking you might angle more of a... I guess you did ask me this. But maybe I didn’t elaborate. There is a big group of people that don’t believe in overboard rescue. There’s a big controversy, I’m sure you know about it. I do both, so I feel justified. But, initially when I started doing the overboard, we were trying to raise awareness in those, for those areas. We would bring awareness to, giant spay and neuter clinics like in Mexico where they would have volunteer vets come down and they do like 2,000 dogs in three days in a certain area. So, we really wanted to educate on spay and neuter and raise the funds for these massive spay and neuter clinics that obviously the people down there have no finances for. So that is important too, it wasn’t just saving that, plucking that one dog out, and bringing it up because we’re never going to… you know we’re not going to rescue all of them. It’s not going to happen in our lifetime. But the education and sterilizing these dogs especially when they have nowhere to go, is, I think, critical. And I know we do that here, but we need to put more effort and feet on the ground and finances and controlling populations elsewhere. So that’s a big part of why I do overboard. Doesn’t mean I raise money for them to do those clinics but more awareness so people can connect and donate and get these things going. It’s still a long way away, like we’re nowhere near but, if I can provide support in that way, that’s just education.

**Follow-up:** Thank you. Yes, that’s definitely something I am interested in exploring in my research and I appreciate you touching on that. Yeah, absolutely you’re right. I am interested in rescue’s motives in that aspect as well, whether they’re aimed more towards education of these areas as well, I know some rescues have a bit more focus on that aspect that they want to educate people, have more foster programs, behaviour, medication, and things like that.

**Participant 8:** Yeah, we do help the other rescues. We’ll send them donations and money to keep going because they are the ones on the ground there. So, I think there’s like a double meaning for me not just to help a dog that may not get the care down there. Knowing that I can find it a home here. But it’s a lot of education too. People have no clue what it’s like sometimes in other places in the world.

**Follow-up:** For other places in the world, did you mean after the dog is rescued?

**Participant 8:** Life on the street… Like when I think of something like Mexico or poor area of Mexico, they’re considered “vermin”, right? You run it over, you run it over. They get hit by a rock, they get hit by a rock. And, you know. I’ve seen dogs macheted, hit with pipes, and have brain damage, So, it’s just stuff that would never fly here, is happening out there and I think. We’re not going to change the world, but it all comes down to spay and neuter. They can’t have that much on the streets and obviously the governments are doing nothing about it. So, I have worked with several groups that do go down and do these massive sterilization projects and that’s kind of the most important thing, I think.

**Follow-up:** Right. Thank you so much for sharing your thoughts on that. With that, I will be concluding the interview.

**P9 Interview Transcript**

**Introductory question:** Can you please remind me, from which geographic areas does your rescue organization import dogs?

**Participant 9:** The mainly- well we rescue dogs- you're just focusing on dogs that are imported right? Because we do work with Northern Ontario and Manitoba, here in Canada mainly. But in regards to importation, most of them come from the US. We have dogs from (South) Korea. We have dogs from Nepal, Kuwait, Iran. Um we're looking at helping dogs right now from Ukraine. We have dogs from Mexico and the Caribbean.

**Question 1:** Wonderful thank you so much, uh, can you tell me how your rescue organization came to the decision to rescuing dogs from these geographical areas? And also I would like to quickly clarify that while my focus is on imported rescue dogs, I would also love to hear about the local experience as well. I find that really interesting.

**Participant 9:** Ok, so I'll start with, I mean rescue from my perspective is something that, I didn't put my hand up, Kai, to be a rescuer, right? When I was a little girl, I remember telling my grandmother I didn't want to have kids. I wanted to have animals because that's something that you have to have in you to be able to do something like this. Um, some people are-come- I mean that has kind of to do with your heart and where your... your calling is and I don't want to sound very weird or spiritual here, but it's every person has a purpose, right? And I knew from the get-go that that was mine. Now with that said, I just came across a post about a dog at some point that needed help and that really opened my eyes to the world of rescue. Um, we're fortunate in Canada that we do not have so many kill rescues, but we do have reserves and the communities up north that do and conduct culls because they don't have the resources to spay and neuter their dogs. The dogs are just there and they're struggling to survive, right? So, the overpopulation, which is the main problem anywhere in Canada and abroad, is what brings this up. The way that the communities up north, whether it's Manitoba or Ontario, deal with this with this is by culling, right? They have shootings, and that's how they control that population. In the US, they have kill-shelters where they euthanize for space. Um, in in the Middle East, it's brutal the way they, those dogs are just on the streets, they don't even have shelters and kids use them for targets and it's just brutal the way they do that. In Asia you have the meat markets right and they capture dogs and that thankfully is getting a lot of attention and it's getting dealt with, but the main thing is that there's an overpopulation of dogs and that they're getting killed right? And that is not resolving the problem, right? That doesn't resolve the problem.

**Follow-up:** Right, thank you so much for elaborating on that. I know there's a lot of discussions online that there's plenty of dogs locally in Canada, and there's a lot of opposition against rescues that import dogs claiming “Why are you bringing in dogs? There's plenty of dogs here” so I'm interested whether it's more of a push situation from other countries where they're in need of help and they're asking these rescues to help locally in Canada, or whether it's more of a pull situation whether there's Canadian rescues that are actively bringing in dogs to Canada. Can you touch on that as well?

Participant 9: Yeah, I can talk about Ontario and I can tell you that humane societies, I mean not rescue groups but regionally funded humane societies, are importing dogs. The [Ontario Humane Society A] brings dogs from [US States], [and Ontario Humane Society B] brings dogs from South Korea because there's not that many dogs here in Ontario, I don't know about other provinces, but in Ontario. Um, the shelters I went to [a neighboring area] to the [local] Humane Society the other day because they donated some food for us. They have four dogs and they're all in fosters, right? So, the demand is greater than the offer here in Ontario. Um, so I think it's a combination of two. There's a lot of people that want to adopt dogs. So that's our perspective. And then there's so many dogs in need, so they're kind of asking us. Every day I get, I don't know thirty, forty, fifty? Asks about dogs that are in the Dominican or are in Mexico or in Texas or in Georgia or whatever that are going to get killed right now because they have no options, right? So.

**Question 2:** Can you tell me about the factors you consider when selecting dogs to rescue?

**Participant 9:** Yes, of course we have to have dogs, our market… and I'm a marketer, that's why I will talk about market. But our market is families. People want a dog that can go on walks or hiking or be there with the family or just a couch potato that can sit and watch TV with them. So, the dogs that we bring, our criteria is pretty simple. It has to be a dog that is dog and people friendly. Right? Because people like to walk their dogs. People don't want the dog that is going to drag them down the street to go and attack another one, so dog and people friendly. Ideally, our ideal dog is one that is good with cats because a lot of people here seem to have cats, so they don't want their cat killed. Um, so dog and people friendly. Um, dogs that are not pit-bulls because we sadly have that ban that we need to abide by or staff terriers. Other than that, dogs that are in the greatest need are the ones that we focus on.

**Follow-up:** Thank you. I'm also curious because you're in this unique position where you also rescue dogs locally. Is there a difference in potentially these factors that you have for local versus imported dogs, or is it more or less the same?

**Participant 9:** It's more or less the same, and what I do… What we cannot import is dogs that are under eight months of age ourselves, right? Because the- we're not allowed. So, a dog that is under the age of eight months has to be picked up on the US side or at the airport by the adopters. Um, with COVID, that was very complicated. This restriction does not apply to dogs that come from Northern Ontario or Manitoba, so we can bring puppies. I mean, there's litters of puppies that are two weeks old, right that are there on the street so we can bring those dogs here, find them a foster, home and we don't have that restriction that we have for dogs that are from outside of Canada.

**Follow-up:** Right, OK, thank you for highlighting that unique difference. This might be a bit of a difficult question, but can you describe to me what a typical dog that you rescue might be, or if there isn't a typical dog that's also fine as well.

**Participant 9:** There's so many. I mean, we rescue dogs that are seniors that are nine years old, right? And they just need a home to live their two or three years that they have left. Um, most of our dogs are adult dogs like between one and five years old dogs in shelters. And on the street they don't last that long, right? So, it's very difficult to find a dog that is older and in need. Unless it's an owner surrender, right? A dog that is surrendered by their owners, but a typical dog is a dog that's dog and people friendly. It's a mutt, I mean a mix of something between one and five, I would say and just... whatever I mean. There are times where we focus on beagle types because those are getting adopted very quickly. Sometimes we focus more on the shepherds. Right, I mean, depends.

**Follow-up:** Got it. Thank you. Um, can you describe to me what life it's like for a dog before it is rescued?

**Participant 9:** Oh my God um. If a dog is in Canada and up north, the dog would be on the streets. Fending for their lives, battling the cold. The dogs tend to live in packs because that's how they survive, and they have to be friendly because they the only food source they have, especially in the winter, comes from people that throw them scraps, right? So that's how they are. Some freeze to death if they're not um in a shelter over the Winter. Um, let's go to Kentucky and dogs are... they come from backyard breeders or just people that don't spay and neuter dogs and some of them sell them. The dogs that are not sold are being just left on the countryside, right? And then animal control is called because they spotted a dog here or there, they're picked up, taken to the shelter. And in the case of the shelters that we work, with um, those dogs are the lucky ones because we will help those that we can. I'll give you an example of Texas where it's not the country like Kentucky. It's a city. San Antonio, and dogs are on the streets, fending from the traffic getting pregnant under sheds or just on a golf course or whatever, picked up by animal control. Animal control kills twice a day and the number of dogs they kill is 30 to 50 every time, right? So those dogs when we pull from that specific shelter, we have 10 minutes. They send you a list of dogs that are being euthanized and you have 10 minutes to blind tag a dog before the dog gets killed. Once you send the tag, you have to pray to God that the dog is still alive, right? Because many times we have received emails saying "Sadly, he's gone," right? And it's devastating. It is devastating. We have had dogs, for example, that are from hoarding situations. Um, there was a lot of coverage maybe four years ago about a house that was, um, the neighbors called Animal Control to a house. They got 40 dogs, 39 dogs in a house. They were dragged out of their homes… The only home that they knew, and the next day, I think that there were 13 left because the rest were euthanized without an assessment, without anything. These dogs were terrified, like they were being pulled up by catch poles on their neck and on their back. It's like-it's just... for us....it is very, very hard to see those things, right? We, in Canada, of those 13 dogs that were spared, two were adopted out locally. One of them was, uh, adopted out. Two were adopted out by a rescue in Michigan. We brought the rest of them here and then the rescue in Michigan transferred one of the dogs to us. So, there's this small Canadian rescue in Canada helping dogs in Texas, right? It's like...the mentality is completely different.

**Follow-up:** Thank you. From talking to also other rescue organizations, they also often mention that there is they have a procedure finding this right match between the dog and the owner. Does your rescue organization also have that, and can you elaborate on that?

**Participant 9:** Yeah, well we have an application form that talks about what is your lifestyle? What is-who are the members of your family? What are you looking for in a dog? And on the other hand, we have assessment on-to each one of our dogs to see how are they with kids, with cats, with other dogs, with people, with males, with females, and so on and so forth. And we try to match them, I mean, for every dog that we post, we might have 3 to 4 applications, so we choose the top two based on the dog. And maybe you are good owner, but you're not my top 2 for that specific dog, but I will find you a dog because we have so many that will match your lifestyle and we will suggest some dogs that might be a good fit for you. We do the reference checks. We talk to the vet. We talk to the landlords if the person is renting and then really try to see. I mean we have placed dogs, there's a bit of controversy right now with, uh, a rescue that said “I'm not adopting a dog out to a family that has an autistic child”. We have done that in the past and it has been successful, but you really need to make sure that the dog is a good match for that family, right? We do meet and greets. We ask them to bring the dog. We let them take the dog as a foster to adopt for 30 days and see how it fits into their house and their lifestyle, so.

**Question 3:** Thank you for elaborating on that. The next question might be more of a sensitive question, but what are some of the costs that are involved in rescuing dogs from abroad or locally?

**Participant 9:** Well, you have come about $250 US to get them vetted and spayed, neutered. We have to do a thorough exam. We need to make sure, that includes fecal and tests and all of that to see if they are heartworm positive or they have Lyme or parasites or whatever, so that has to be done. The dogs that are over six months of age or spayed or neutered and the cost it depends on where it is, but it's around $250 US. The cost of transportation of a dog to Canada is between 150 and $175 US. And then you have to import the dog, right? So, you have to pay duties, you have to pay a broker and that. So, I would say that it is about between 400... about $450 US, which is about $600 Canadian, more or less. If the dog goes into a foster to adopt home, that's fine. Our adoption fee is $650, but sometimes the dog has to go into a kennel while we find an available foster, so we need to pay $30 per dog per day for them to be in a kennel. Sometimes they need a follow up vet appointment. So, we're not for profit, so that's that. There's dogs that come that have come that need follow up surgeries. We have dogs with heart murmurs. We have dog that has an immune- autoimmune disease so his fur is it's almost like he has mange, but it's not mange. There’s dogs that have pellets, that-they have been shot at, so we need to have- they need to have surgeries and stuff right? So, the surgeries will go from $8,000 to $9,000 down to maybe $2,000 and that has to be covered by fundraisers, right? Because the vet-the adoption fees will not cover that.

**Follow-up:** Yeah, that was actually my follow-up question, apart from fundraisers, how do you recuperate these costs? You also mentioned adoption fees, but is there anything else that you do in particular?

**Participant 9:** Yeah, we do fundraisers. We have a donor base right now that is growing and we have events we haven't been able to do events, that's why everybody is struggling right now, right? To have funds. But with Covid we were unable to have events. We're hopefully going to be able to do that um, starting in the summer, we'll see.

**Follow-up:** Right, thank you. Has there ever been a time where you couldn't rescue a dog because it was too expensive?

**Participant 9:** Oh yeah, yes we have, um, and we try not to say no to a dog, but um. But there's times when you know the dog is either... you know what? I don't know that it would be... I have a dog that we ended up rescuing, um, and she had a Grade 4 heart murmur so we said we're sorry we can't. It's $300 a month for the medication and one of our volunteers said I want her. I will cover her so we asked her to do it directly, right? Rather than us getting in the middle, so we said no, but she was rescued by this volunteer that wanted to take that on, right? I can say, and I can give you a number of examples of dogs that we have. I mean we got a call from a vet in Kentucky saying a good Samaritan found a dog on the side of the road. She was hit by a car. She's a Great Pyrenees. He brought her to the clinic. He's going to chip-in and she needs extensive surgery, so he's going to give $1,000 towards the surgery. The vet is going to cover $1,000, and would you help the dog and I'm like “so what are we talking about?” So, it was $3,500 so we said OK yeah, we’ll help her. And we did. Then she came here, and she needed tumor surgeries, right? And that was about $8,000 and change that we had to cover for her, but for us, it's not about making money, it's about helping those that need it, right? And that's why we helped her, and she ended up being adopted by the person in Kentucky that found her, so we had to transport her back to Kentucky um to this family and they're wonderful with her, right? And that's what we want for every dog that we have.

**Question 4:** Tell me about some challenges you face through your work? I'm sure there's plenty. Some major ones would be great.

**Participant 9:** I think the biggest challenge is, yeah, I'll say two and it comes down to people. Um, one of the things that we're going to be working on, Kai, and let me just show you here like this, is my computer, right? And it has all this post-it notes and of what I need to work on. But it's responsible dog ownership. When you own a dog, its considered property right? And let's just think of a, uh... You buy a car, right? A used car, and the car, in two weeks, has a problem. What we have is that people turn around and say, oh, you sold me a used car and it's defective. Now, you have to pay for it. We don't have the money to do that. We have these dogs fully vetted. I mean this example that I gave you about Sweetie, the dog, the Great Pyrenees. She had a family when she came here. And she went to the family and the family called us within a week and said “I don't want to be changing the bandages because she had some bandages. So, I want to take her to the vet to change the bandages. That's $100 every time I go to the vet. Can you pay for that?” And we’re like, you adopted this dog. So, either return the dog to us and we will take care of her medical needs, or you need to be responsible for that. We cannot be paying for the dogs in perpetuity, like that's not how it works, right? You have a kid and the kid is sick, and now do you return to kid? Like, what are you doing? So, it’s responsible dog ownership, that's one of the challenges, right? For people to take ownership, or we have had situations where “oh, I had the dog for a year. And now I have a new boyfriend and my dog doesn’t like the boyfriend”. It’s like... so what are you going to do about it? Like this dog is yours. He has lived your life with you. He's part of your family. Now you're returning the dog because your boyfriend doesn't like the dog? And there's some... so that is, I think the biggest challenge.

The other challenge is… and I will call this as it is. There's a group of people that are in rescue limbo. So, people that have volunteered with us, then went to another rescue. They got kicked out of that rescue. They went to another one and they get kicked out because they're very negative and very nasty, right? And these people are now taking on calling out all dog rescues because they are doing wrong, or they're doing whatever. It's a group of this rescuers that have really no home, because nobody wants them, and smaller rescues that, instead of putting their heads down and doing the work that you need to do to be successful, are looking at what others are doing and try to bring them down. That, I have no use for that.

**Follow-up:** So just to clarify, those are other rescues.

**Participant 9:** Smaller rescues that haven't been able to take off, right?

**Follow-up:** Thank you for elaborating that. I've also heard some similar concerns as well raised by other rescues. This might be a difficult question as well, but what can be done to possibly reduce or eliminate these challenges, if there is anything that could be done?

**Participant 9:** We're going to be launching… And that's why I showed you my post-its. I'm going to be launching an education campaign in regards to responsible dog ownership, right? Educating people on what it really means to be a responsible dog owner, right? It's… the dog is a living, breathing, sentient being that is a part of your family. They will only learn what you teach them, right? So, it's your responsibility to show the boundaries, what is there to be done, and so on. In regards to those people that are posting very nasty stuff online, our work speaks for itself, right? I mean… And that's what I've said to my volunteers. We are not going to be attacked. I don't care what other rescues are doing, I want to move us forward and we're moving forward and that's why we have been in business for seven years, successfully. That's why we have been able to place the number of dogs that we have. Um, so we're not interfering or interested in what other rescues are doing. We're minding our own business, so I think that what we're going to be doing is showing our work a little more, um, and highlighting all the good that we do, right? Because that in and of itself is going to take care of… I mean, one of the things that that these people are saying is that rescue is unregulated. Well, it isn't unregulated. We need to comply with- first of all, we have to be a registered business. Then we need to comply with the CRA (Canadian Revenue Agency), in regard to taxes. We are a registered charity, so we were audited. Our financials are audited every year when we submit our taxes. We have to comply with importation rules set out by the CBSA (Canadian Border Services Agency). We need to comply with transportation rules and the health of the animals that we’re bringing here and those are set by the CFIA (Canadian Federal Inspection Agency), so it's not like we're putting dogs in the back of a pick-up and going across the border and selling them on the streets, right? So anyways.

**Preamble:** From what you’ve described during this interview, it must be very challenging and demanding to work in a rescue organization.

**Question 5:** Can you please tell me what it is that keeps you continuing your work?

**Participant 9:** It's my calling. I'll go back to my original, um, statement. It is...my purpose. I am a mother. I am a marketer, I'm a wife. I have one son that is 20 years old in university. He plays baseball and he is in [US State], so. We're empty nesters, right and, you know, my son has not needed mommy like he needed me when he was growing up. So, um, this is what I do, right? And there's so much need out there, and on the other hand, if I look at what we have been able to accomplish, we have rescued over 7,000 dogs in seven years. Um, we have placed them in homes, they're thriving in their homes. Some have died of old age or because they had an illness later on, but they lived happy lives, right? And that's what we work for.

**Follow-up:** Thank you. What do you enjoy most in your work?

**Participant 9:** Seeing dogs thrive in their lives, and seeing my volunteers enjoy and celebrate each one of these placements, right? Yeah, I have my own dogs. Well, they... I think one of the greatest gifts you can give anybody is really a dog to a family, right? Because they will be loyal and bring you joy and they're silly and they're like toddlers, right?

**Follow-up:** Thank you. In in a perfect world, what would the lives of all dogs look like for you?

**Participant 9:** Like mine. Like they would be like my dogs. With a family, um, what I always say is that what we wish for a dog is the safety of a home and the love of a family. That's what it would look like in a perfect world. Every dog would have that, the safety of a home and the love of a family.

**Preamble:** I’ve asked you a lot of questions today, and I want to thank you once again for sharing your thoughts and experiences with me.

**Question 6:** Before I conclude this interview, I’m wondering if there’s anything I haven’t asked you about that you think it is important for me to know?

**Participant 9:** Hmm...I don't know. I'm looking at my little post-it notes here.

**Follow-up:** Yeah, no worries. Yeah I have plenty of time so if you need a bit of time to think about anything, please take your time.

**Participant 9:** Yeah no I don't...No, I cannot think of anything.

**Follow-up:** That's great, yeah, if you do, maybe later on think something, remember of something that might be urgent you can always contact me on email and that's wonderful. OK, great then that's all the questions that I had, so we actually burned through this interview really pretty fast. So, thank you once again for agreeing to participate. I have a quick speech prepared at the end just to wrap up the interview, so I'll just quickly do that
